# Coordination-Driven Self-Assembly of Silver(I) and Gold(I) Rings: Synthesis, Characterization, and Photophysical Studies

**DOI:** 10.3389/fchem.2019.00567

**Published:** 2019-08-13

**Authors:** Cressa Ria P. Fulong, Sewon Kim, Alan E. Friedman, Timothy R. Cook

**Affiliations:** ^1^Department of Chemistry, University at Buffalo, The State University of New York, Buffalo, NY, United States; ^2^Department of Materials Design and Innovation, University at Buffalo, The State University of New York, Buffalo, NY, United States

**Keywords:** self-assembly, coordination, metallacycle, silver hexagon, gold hexagon

## Abstract

In this work, we investigate the self-assembly between Ag(I) and Au(I) centers and pyridyl donors to form hexagonal metallacycles and related linear complexes. The precipitation of hexagonal metallacycles upon assembly in chloroform/methanol mixtures results in high solid-state photo-stability. Whereas, the Ag(I) species have fast kinetics and high formation constants in acetone, this solvent interferes in the formation of the analogous Au(I) complexes. The photophysical properties of this suite of metallacycles was investigated including steady-state absorption, emission, and time-resolved lifetime measurements. All ligands and hexagons exhibited ligand-centered singlet emissions with ground-state absorption and emission perturbed upon coordination. The ligand-based fluorescent photoluminescence was affected by the heavy-atom effect when halide or metals are present, attenuating quantum yields as evidenced by increases in the experimentally measured non-radiative rate constants. The formation of group 11 metallacycles is motivated by their potential applications in mixed-matrix materials wherein metal ions can interact with substrate to facilitate separations chemistry with reduced energy requirements, in particular the isolation of ethylene and light olefins. Existing processes involve cryogenic distillation, an energy intensive and inefficient method.

## Introduction

Coordination-driven self-assembly (Leininger et al., 2000) is a valuable strategy for the design and synthesis of discrete molecular complexes such as metallacycles and metallacages for biomedical (Cook et al., 2013; Casini et al., 2017) catalytic (Ito et al., 2000; Brown et al., 2009; Smulders and Nitschke, 2012; Vardhan and Verpoort, 2015; Kuijpers et al., 2016), molecular sensing (Tashiro et al., 2005; Kim and Ahn, 2008; Wang et al., 2011; Zwijnenburg et al., 2016; Cao et al., 2017), and small molecule adsorption and separation applications (Sudik et al., 2005; Zhao et al., 2010; Riddell et al., 2011; Amayuelas et al., 2016). This strategy exploits pre-programmed directionality information on metal acceptor and ligand donor building blocks to form complex architectures driven by metal-ligand coordination (Cook and Stang, 2015). A careful design of building blocks coupled with thermodynamic driving forces favors the convergent formation of discrete molecular complexes over divergent coordination polymers a.k.a. metal-organic frameworks (MOFs) (Fujita et al., 2001).

Inspired by previous reports on hexagonal self-assemblies from *trans*-capped Pt(II) precursors (Yang et al., 2007) and 120° bidentate pyridine-type ligands, which are known for their luminescent properties, and other luminescent Pt(II)-based metallacycles (Huang et al., 2017; Tang et al., 2018), we now report the self-assembly of hexagonal metallacycles from the same class of ligand donors and Ag(I) and Au(I) metal acceptors. There have been few reports on linear Ag(I) (Del Piero et al., 2008) and Au(I) (Fernández et al., 2007) complexes, and Ag(I) metallacycles (Shin et al., 2003; Chen and Mak, 2005; Fromm et al., 2005; Ren et al., 2006; Kim et al., 2009; Wan and Mak, 2011; Kole et al., 2012; Wei et al., 2012; Chevrier et al., 2013) with pyridine-type ligands, but these are the first Ag(I) and Au(I) hexagonal rings. We also synthesized Ag(I) and Au(I) monomeric complexes using analogous monodentate pyridine-type ligands. Having both linear monomeric complexes and hexagonal metallacycles enables the comparison of solid- and solution-state structures, organic solvent solubilities, photophysical properties, as well as light-stabilities. These self-assembled Ag(I) and Au(I) hexagons have limited solubility in organic solvents. Efforts are ongoing to improve the solubilities of these metallacycles including but not limited to pre- and post-assembly ligand modification.

We have an ongoing interest in incorporating these metallacycles and metallacages as filler materials in mixed-matrix materials (MMMs) (Fulong et al., 2018). MMMs are a type of thin-film composite material typically composed of a highly porous inorganic/organic filler and an organic polymer binder. The significance of MMMs in the medical and small molecule purification industries has been steadily increasing in the past decade and contemporary research is largely focused on MOFs as a filler material (Dechnik et al., 2017). We have previously shown the importance of solubilizing the filler material into the polymer solution to fabricate thin, flexible, homogeneous, and highly permeable MMMs (Fulong et al., 2018). Along these lines, we highlighted the advantage and ease of using discrete coordination cages over MOFs as MMMs filler material due to the former's solubility in a range of solvents. For certain separations the filler material introduces sites for intermolecular interactions with substrate, which enables active separation rather than just size-selective sieving. As a result, the design of fillers with unsaturated metals or other binding sites enables the fabrication of MMMs with improved permeability and selectivity. Since a variety of metal centers may be used as nodes in coordination-driven self-assembly, properly matching metal ions to a separation of interest is an important first-principle in the design of new metal-organic materials (Dechnik et al., 2017).

The incorporation of silver salts has been identified as an effective strategy for increasing the selectivity of olefin/paraffin membranes based on the ability of electron-rich olefins to interact with metal ions, an attractive technology given the scope of the petrochemical industry wherein ethylene is used a primary building block (Eldridge, 1993). These materials suffer from the instability of Ag(I) ions that react with oxygen and leach from the material. We hypothesized that Ag(I) ions may be stabilized by a linear L–Ag(I)–L environment that still enables equatorial interactions with substrate (Fox et al., 2002; Gimeno, 2009). Other transition metals with coordination numbers of three or more require capping ligands to prevent divergent framework formation (Fujita, 1998; Fujita et al., 2001). For Ag(I) and Au(I), with linear coordination environments, capping ligands are not required and coordination with bent ditopic donors may furnish discrete metallacycles.

## Materials and Methods

All reagents and solvents were reagent grade and used as received without further purification unless noted. Methanol (CH_3_OH), acetone, dichloromethane (DCM), ethyl acetate (EtOAc), n-hexanes, toluene, diethyl ether, petroleum ether, potassium hydroxide (KOH), sodium sulfate (Na_2_SO_4_) and potassium carbonate (K_2_CO_3_) were purchased from Fisher Scientific. N,N-dimethylformamide (DMF) was purchased from Macron Fine Chemicals. Tetrahydrofuran (THF), triethylamine (NEt_3_), and chloroform (CHCl_3_) were purchased from EMD Millipore. Ethanol (EtOH, 200 proof) was purchased from Decon Labs, Inc. Tetrakis(triphenylphosphine)palladium(0) (Pd(PPh_3_)_4_), and bis(triphenylphosphine)palladium(II) chloride (Pd(PPh_3_)_2_Cl_2_), and 1,3-dibromobenzene were purchased from Matrix Scientific. 4-pyridinylboronic acid (pyB(OH)_2_) was purchased from AK Scientific. Copper (I) iodide (CuI) was purchased from Strem Chemicals. Tetrahydrothiophene (tht) was purchased from Aldrich. Silver(I) hexafluorophosphate (AgPF_6_) was purchased from Oakwood Chemical. Hydrogen tetrachloroaurate(III) trihydrate (HAuCl_4_·3H_2_O) was purchased from J&J Materials Inc. 4-ethynylpyridine hydrochloride and 1,3,5-tribromobenzene were purchased from Ark Pharm Inc. Deionized water was used whenever water was needed. THF was purified and dried through a Pure Process Technology free standing solvent purification system. NEt_3_ was purified and dried by distillation in KOH. DMF was filtered and dried using activated molecular sieves under N_2_ and freeze-pump-thawed with water before use for synthesis.

^1^H Nuclear Magnetic Resonance (NMR) spectra were recorded on either Varian 300 or 400 MHz spectrometer. Chemical shifts (δ) are reported in parts per million (ppm) referenced using the residual protio-solvent peaks as internal standards. Coupling constants (J) are quoted in Hertz (Hz), and the following abbreviations are used to describe the signal multiplicities: s (singlet); d (doublet); t (triplet); q (quartet); m (multiplet). Fourier Transform-Ion Cyclotron Resonance (FT-ICR) mass spectra were acquired using a Bruker Daltonics SolariX 12T FT-ICR Mass Spectrometer that was calibrated with > 90% Angiotensin I purchased from Sigma Aldrich under either Electrospray Ionization (ESI) or Laser Desorption Ionization (LDI) modes. Single Crystal X-ray Diffraction (SC-XRD) Crystallography was performed using a Bruker D8 Venture diffractometer in a fixed-chi geometry, equipped with a Photon-100 CMOS area detector, Oxford Cryosystems cryostat, a molybdenum source (Mo, λ = 0.71073 Å), and a graphite monochromator. Fourier Transform Infrared Resonance (FT-IR) spectra were collected from a Perkin Elmer 1760 FT-IR spectrometer equipped with horizontal attenuated total reflectance (HATR) from 4,000 to 500 cm^−1^ wavenumbers (ν). The following abbreviations are used to describe signal intensities: w (weak); m (medium); s (strong). All UV-Vis absorption spectra were acquired from an Agilent Cary 8454 UV-Vis Diode Array system. Blank spectra with pure solvents were acquired before each run. All emission spectra were collected using a Horiba Scientific FluoroMax-4 Spectrofluorometer equipped with Quanta-φ Integrating light sphere with Spectralon coating for quantum yield (Φ) measurements and Delta-Hub DH-HT High Throughput Time-correlated Single Photon Counting (TCSPC) Controller and Nano-LED NL-C2 pulsed-diode controller with N-350 NanoLED Source for lifetime measurements. All solution-state absorbance and emission spectra were collected using a 10-mm rectangular quartz cuvette from Starna Cells Inc.

### Synthesis of Ag(I) Complex (AgL1_2_)

Ag**L1**_2_ complex was self-assembled using a modified literature procedure (Chen et al., 2013). In a foil-covered 200-mL round-bottom flask, 0.800 g (3.1 mmol) **L1** was dissolved in 80.0 mL CHCl_3_. In another foil-covered 100-mL round-bottom flask, 0.720 g (2.85 mmol) AgPF_6_ was dissolved in 80.0 mL MeOH then layered over the **L1** solution. After 48 h, white needle-like crystals form. These crystals were filtered and washed with 10-mL portions of CHCl_3_ (3x) to afford 1.19 g (100% yield) isolated product. ^1^H NMR (400 MHz, acetone-d_6_, 25°C): δ (ppm) = 8.81 (d, ^3^J = 5.2 Hz, 4H, pyridyl H_α_), 7.82 (s, 2H, phenyl H), 7.77 (d, ^3^J = 6.3 Hz, 4H, pyridyl H_β_), 7.71 (d, ^3^J = 8.1 Hz, 2H, phenyl H), 7.65 (d, ^3^J = 7.8 Hz, 2H, phenyl H), 7.46 (t, ^3^J = 7.9 Hz, 2H, phenyl H). FT-IR (ATR, cm^−1^): 3069 (w), 2235 (w), 2187 (w), 1609 (m), 1558 (w), 1506 (w), 1470 (w), 1433 (w), 1405 (w), 1223 (w), 1152 (w), 1070 (w), 1031 (w), 991 (w), 915 (w), 881 (m), 815 (m), 775 (m), 757 (m), 743 (m), 678 (m), 663 (m), 553 (m), 531 (m).

### Synthesis of ({AgL2}_6_) Ag(I) Hexagon

{Ag**L2**}_6_ hexagon, was self-assembled using a modified literature procedure (Chen et al., 2013). In a foil-covered 20-mL scintillation vial, 0.050 g (0.14 mmol) **L2** was dissolved in 5.0 mL CHCl_3_. In another foil-covered 20-mL scintillation vial, 0.035 g (0.14 mmol) AgPF_6_ was dissolved in 5.0 mL MeOH then layered over the **L2** solution. After 48 h, white powdered product formed which was filtered and washed with 10-mL portions of CHCl_3_ (3x) to afford 0.062 g (73% yield) isolated product. ^1^H NMR (400 MHz, acetone-d_6_, 25°C): δ (ppm) = 8.72 (d, ^3^J = 5.9 Hz, 24H, pyridyl H_α_), 7.90 (s, 11H, phenyl H), 7.83 (s, 6H, phenyl H), 7.62 (d, ^3^J = 6.0 Hz, 24H, pyridyl H_β_). FT-IR (ATR, cm^−1^): 3076 (w), 2219 (w), 1613 (m), 1557 (w), 1507 (w), 1435 (m), 1338 (w), 1291 (w), 1221 (m), 1174 (w), 1162 (w), 1136 (w), 1107 (w), 1066 (w), 1028 (w), 993 (w), 964 (w), 864 (m), 821 (s), 748 (w), 736 (w), 663 (m), 573 (w), 552 (s).

### Synthesis of ({AgL3}_6_) Ag(I) Hexagon

{Ag**L3**}_6_ hexagon, was self-assembled using a modified literature procedure (Chen et al., 2013). In a foil-covered 20-mL scintillation vial, 0.050 g (0.18 mmol) **L3** was dissolved in 5.0 mL CHCl_3_. In another foil-covered 20-mL scintillation vial, 0.045 g (0.18 mmol) AgPF_6_ was dissolved in 5.0 mL MeOH then layered over the **L3** solution. After 48 h, white powdered product formed which was filtered and washed with 10-mL portions of CHCl_3_ (3x) to afford 0.096 g (100% yield) isolated product. ^1^H NMR (400 MHz, acetone-d_6_, 25°C): δ (ppm) = 8.74 (d, ^3^J = 6.0 Hz, 24H, pyridyl H_α_), 7.86 (s, 6H, phenyl H), 7.74 (d, ^3^J = 9.3 Hz, 12H, phenyl H), 7.66 (d, ^3^J = 6.1 Hz, 23H, pyridyl H_β_), 7.63 – 7.56 (m, 7H, phenyl H). FT-IR (ATR, cm^−1^): 3107 (w), 2203 (w), 1615 (m), 1543 (w), 1501 (w), 1431 (w), 1227 (w), 1167 (w), 1064 (w), 1025 (w), 878 (w), 821 (m), 752 (w), 733 (w), 682 (w), 556 (m), 541 (m).

### Synthesis of ({AgL4}_6_) Ag(I) Hexagon

{Ag**L4**}_6_ hexagon, was self-assembled using a modified literature procedure (Chen et al., 2013). In a foil-covered 20-mL scintillation vial, 0.025 g (0.11 mmol) **L4** was dissolved in 2.0 mL CHCl_3_. In another foil-covered 20-mL scintillation vial, 0.027 g (0.11 mmol) AgPF_6_ was dissolved in 2.0 mL MeOH then layered over the **L4** solution. After 48 h, white powdered product formed which was filtered and washed with 10-mL portions of CHCl_3_ (3x) to afford 0.052 g (92% yield) isolated product. ^1^H NMR (400 MHz, acetone-d_6_, 25°C): δ (ppm) = 8.76 (d, ^3^J = 6.7 Hz, 24H, pyridyl H_α_), 8.25 (s, 5H, phenyl H), 8.00–7.87 (m, 36H, pyridyl H_β_ and phenyl H), 7.80–7.71 (m, 5H, phenyl H). FT-IR (ATR, cm^−1^): 3092 (w), 1618 (m), 1545 (w), 1513 (w), 1478 (w), 1431 (w), 1411 (w), 1314 (w), 1230 (w), 1069 (w), 1031 (w), 1019 (w), 873 (m), 818 (s), 791 (s), 736 (m), 695 (m), 663 (w), 637 (m), 613 (w), 555 (s).

### Synthesis of Au(I) Complex (AuL1_2_)

Au**L1**_2_ complex was self-assembled using a modified literature procedure (Lin et al., 2008). In a foil-covered 2-dram vial, 0.020 g (0.078 mmol) **L1**, 0.025 g (0.078 mmol) Au(tht)Cl were dissolved in 6.0 mL acetone. After 6 h, the solution was concentrated in vacuo and the collected solid was washed with 6-mL portions of CHCl_3_ (3x) to afford 0.007 g (20% yield) isolated product. ^1^H NMR (300 MHz, acetone-d_6_, 25°C): δ (ppm) = 8.84 (d, ^3^J = 6.0 Hz, 4H, pyridyl H_α_), 7.89 (d, ^3^J = 7.0 Hz, 5H, pyridyl H_β_), 7.80 (s, 2H, phenyl H), 7.68 (td, ^3^J = 16.3, 15.7, 7.8 Hz, 10H), 7.50 – 7.40 (m, 3H, phenyl H). FT-IR (ATR, cm^−1^): 3044 (w), 2918 (w), 2856 (w), 2218 (m), 2187 (w), 1943 (w), 1801 (w), 1614 (m), 1588 (m), 1558 (w), 1496 (w), 1463 (w), 1429 (w), 1404 (w), 1261 (w), 1234 (w), 1217 (w), 1158 (w), 1105 (w), 1088 (w), 1060 (w), 1038 (w), 987 (w), 906 (w), 876 (w), 836 (m), 814 (w), 789 (m), 747 (w), 713 (w), 691 (w), 680 (m), 655 (m), 565 (w), 535 (m).

### Synthesis of Au(I) Hexagons ({AuL2}_6_)

{Au**L2}**_6_ hexagon was self-assembled using a modified literature procedure (Lin et al., 2008). In a foil-covered 2-dram vial, 0.028 g (0.078 mmol) **L2**, 0.025 g (0.078 mmol) Au(tht)Cl were dissolved in 6.0 mL CHCl_3_. After 24 h, the solution was concentrated in vacuo and the collected yellow solid was washed with 6-mL portions of CHCl_3_ (3x) to afford 0.025 g (78% yield) isolated product. ^1^H NMR (400 MHz, CD_3_NO_3_, 25°C): δ (ppm) = 8.84–8.71 (m, 24H, pyridyl H_α_), 8.09 – 7.82 (m, 40H, pyridyl H_β_ and phenyl H). FT-IR (ATR, cm^−1^): 3100 (w), 3044 (w), 2210 (m), 1615 (s), 1553 (w), 1496 (w), 1430 (m), 1336 (w), 1288 (w), 1211 (m), 1171 (w), 1134 (w), 1105 (w), 1063 (m), 1046 (w), 992 (w), 966 (w), 863 (m), 826 (m), 761 (w), 721 (w), 672 (m), 587 (w), 574 (m), 527 (w).

### Synthesis of Au(I) Hexagons ({AuL3}_6_)

{Au**L3**}_6_ hexagon was self-assembled using a modified literature procedure (Lin et al., 2008). In a foil-covered 2-dram vial, 0.022 g (0.078 mmol) **L3**, 0.025 g (0.078 mmol) Au(tht)Cl were dissolved in 6.0 mL CHCl_3_. After 24 h, the solution was concentrated in vacuo and the collected yellow solid was washed with 6-mL portions of CHCl_3_ (3x) to afford 0.026 g (89% yield) isolated product. ^1^H NMR (400 MHz, CD_3_NO_3_, 25°C): δ (ppm) = 8.76 (d, ^3^J = 5.0 Hz, 24H, pyridyl H_α_), 8.08 – 7.70 (m, 41H, pyridyl H_β_ and phenyl H), 7.66–7.57 (m, 7H, phenyl H). FT-IR (ATR, cm^−1^): 3092 (w), 3029 (w), 2203 (m), 1943 (w), 1609 (s), 1529 (w), 1492 (w), 1427 (w), 1324 (w), 1213 (w), 1162 (w), 1150 (w), 1125 (w), 1093 (w), 1065 (w), 1042 (w), 991 (w), 942 (w), 902 (w), 831 (m), 792 (w), 754 (w), 729 (w), 666 (w), 569 (w), 544 (w).

### Synthesis of Au(I) Hexagons ({AuL4}_6_)

{Au**L4**}_6_ hexagon was self-assembled using a modified literature procedure (Lin et al., 2008). In a foil-covered 2-dram vial, 0.018 g (0.078 mmol) **L4**, 0.025 g (0.078 mmol) Au(tht)Cl were dissolved in 6.0 mL CHCl_3_. After 24 h, the solution was concentrated in vacuo and the collected white solid was washed with 6-mL portions of CHCl_3_ (3x) to afford 0.027 g (100% yield) isolated product. ^1^H NMR (400 MHz, CD_3_NO_3_, 25°C): δ (ppm) = 8.82 (d, ^3^J = 6.7 Hz, 24H, pyridyl H_α_), 8.35 (s, 7H, phenyl H), 8.22 (d, ^3^J = 6.3 Hz, 22H, pyridyl H_β_), 8.11 (d, ^3^J = 9.4 Hz, 14H, phenyl H), 7.86 (t, ^3^J = 7.6 Hz, 7H, phenyl H). FT-IR (ATR, cm^−1^): 3060 (w), 1611 (s), 1546 (w), 1508 (w), 1472 (w), 1443 (w), 1427 (w), 1398 (m), 1340 (w), 1318 (w), 1266 (w), 1231 (m), 1186 (w), 1109 (w), 1076 (m), 1034 (w), 970 (w), 935 (w), 915 (w), 857 (w), 832 (m), 786 (s), 761 (w), 732 (m), 687 (w), 667 (w), 645 (m), 632 (w), 606 (w), 535 (s).

## Results and Discussion

### Self-Assembly of Ag(I) and Au(I) Complexes and Hexagons

**Scheme 1:**
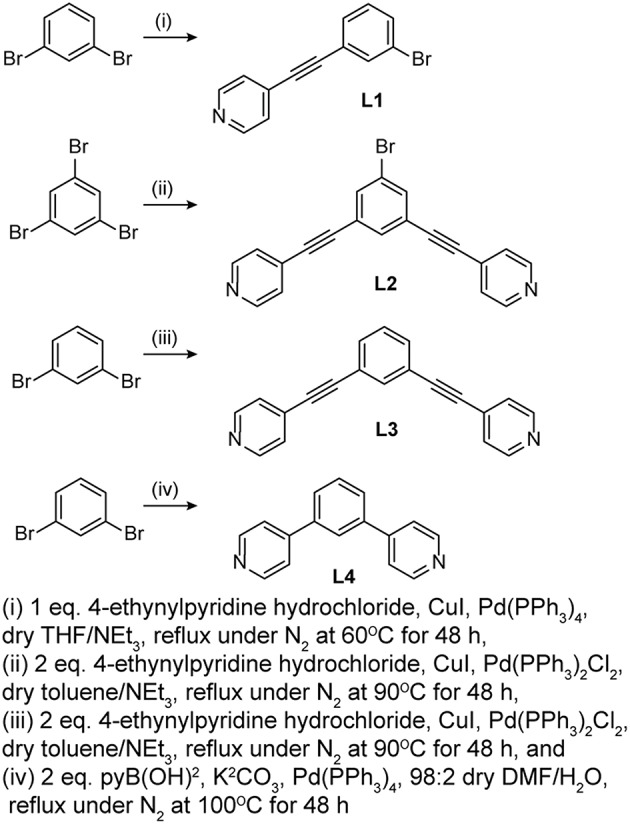
Coupling reactions to form **L1**, **L2**, **L3**, and **L4**.

Four pyridyl ligands, 1-bromo-3(4-ethynylpyridyl)benzene (**L1**), 5-bromo-1,3-bis(4-ethynylpyridyl)benzene (**L2**), 1,3-bis(4-ethynylpyridyl)benzene (**L3**), and 1,3-bis(4-pyridyl)benzene (**L4**), were prepared as donors for self-assembly reactions. As illustrated in Scheme 1, **L1**, **L2**, and **L3** were prepared following a typical Sonogashira coupling reaction while **L4** was prepared following a typical Suzuki coupling reaction. The ^1^H NMR spectra of **L1**, **L2**, **L3**, and **L4** in acetone-d_6_ are summarized in Supplementary Figures 1A–D, respectively. The **L1** ^1^H spectrum shows the pyridyl-H doublets at 8.63 and 7.49 ppm and the benzyl-H singlet at 7.78 ppm, two doublets at 7.65 and 7.60 ppm, and triplet at 7.42 ppm. The **L2** spectrum shows the pyridyl-H doublets at 8.66 and 7.53 ppm and the benzyl-H singlets at 7.87 and 7.82 ppm. The **L3** spectrum shows the pyridyl-H doublets at 8.65 and 7.51 ppm and the benzyl-H singlet at 7.83 ppm, doublet at 7.69 ppm, and triplet at 7.56 ppm. The **L4** spectrum shows the pyridyl-H doublets at 8.68 and 7.78 ppm and the benzyl-H singlet at 8.17 ppm, doublet at 7.89 ppm, and triplet at 7.70 ppm.

**Figure 1:**
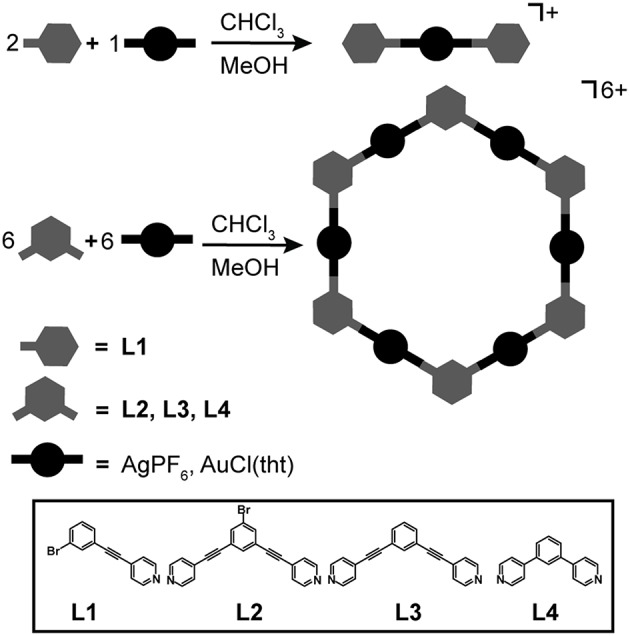
Self-assembly of Ag(I) and Au(I) hexagons and mononuclear homoleptic complexes.

**L1** was used to synthesize linear Ag(I) and Au(I) complexes, while **L2**, **L3**, and **L4** were used to self-assemble Ag(I) and Au(I) hexagonal metallacycles. The general method used to self-assemble the complexes and hexagons is shown in Figure 1. All self-assembly reactions were performed in the absence of light to ensure no loss in Ag(I) and Au(I) precursors. The monomeric Ag(I) complex, Ag**L1**_2_, was prepared by layering one equivalent of AgPF_6_ in MeOH over two equivalents of **L1** in CHCl_3_. This resulted in the crystallization of Ag**L1**_2_ after 48 h. Ag(I) hexagons, {Ag**L**}_6_ where **L** = **L2**, **L3**, and **L4**, were prepared by layering one equivalent of AgPF_6_ in MeOH over one equivalent of **L** in CHCl_3_. The hexagons precipitated at almost quantitative yields as white powders after 48 h. The gold analogs, Au(I) hexagons, {Au**L**}_6_ where **L** = **L2**, **L3**, and **L4**, were prepared by mixing 1:1 equivalents of Au(tht)Cl and **L** in CHCl_3_. After 24 h, powdered metallacycles were isolated upon solvent removal and CHCl_3_ washing. In the case of the monomeric Au(I) complex, Au**L1**_2_, previous attempts using pure CHCl_3_ or 1:1 (v/v) CHCl_3_/MeOH did not result in any complex formation. However, the complex was isolated at a relatively low yield by mixing 1:1 equivalents of Au(tht)Cl and **L1** in acetone.

### ^1^H NMR of Ag(I) and Au(I) Complexes and Hexagons

A notable challenge in characterizing these self-assembled Ag(I) and Au(I) complex and hexagons is their limited solubilities. Coordinating solvents such as acetonitrile (MeCN) and *N,N'*-dimethylsulfoxide (DMSO), which are typically used in solution-state structural analyses of Ag(I) and Ag(I) complexes proved to be unsuitable for our complexes and hexagons (Laye, 2007; Lin et al., 2008). In all cases, we observed ligand dissociation upon addition of MeCN or DMSO.

**Figure 2:**
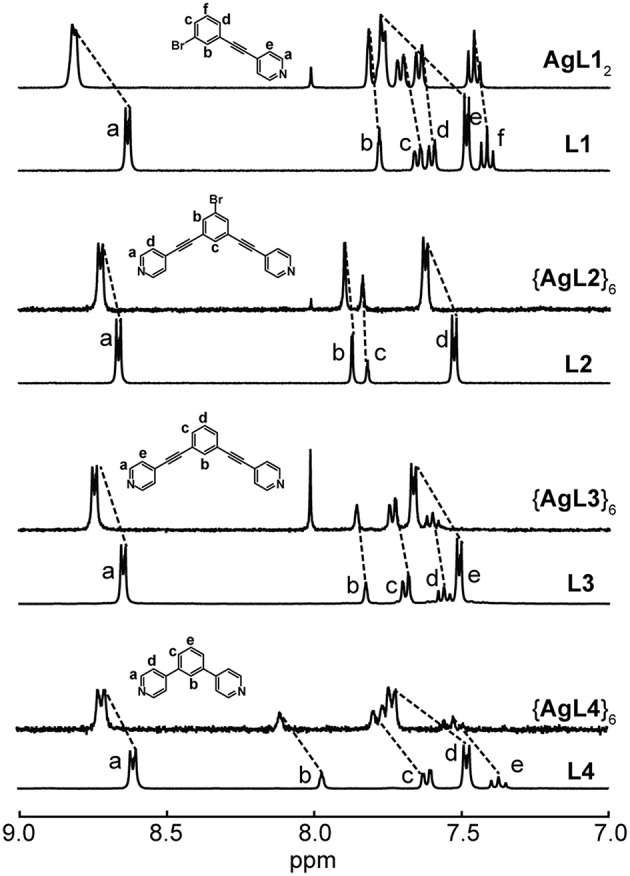
^1^H NMR spectra of **L1** and Ag**L1**_2_, **L2** and {Ag**L2**}_6_, **L3** and {Ag**L3**}_6_ and **L4** and {Ag**L4**}_6_ in acetone-d_6_.

**Figure 3:**
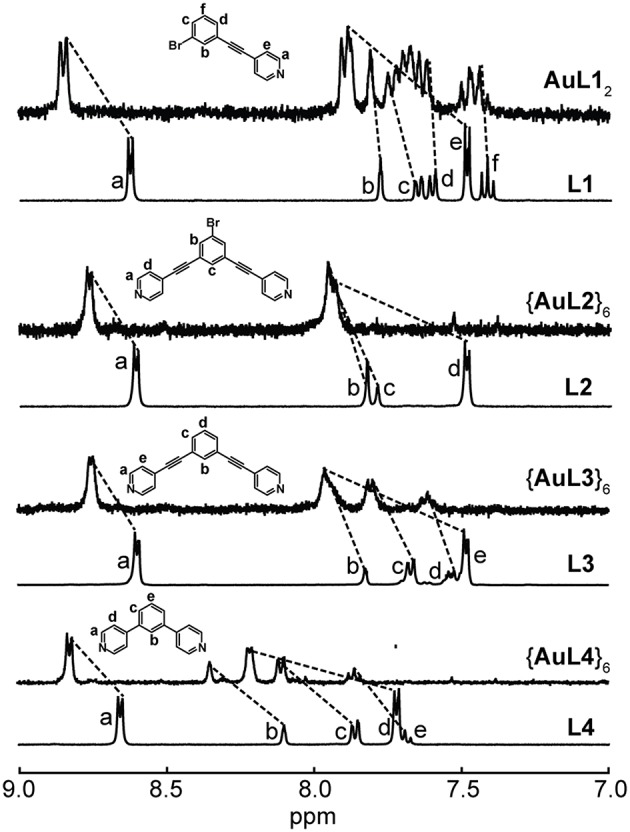
^1^H NMR spectra of **L1** and Au**L1**_2_ in acetone-d_6_, **L2** and {Au**L2**}_6_, **L3** and {Au**L3**}_6_ and **L4** and {Au**L4**}_6_ in nitromethane-d_3_.

Nonetheless, we were able to detect the intact Ag(I) complexes and hexagons (Figure 2) as well as Au(I) complex (Figure 3) in acetone. The coordination of the pyridyl ligands to Ag(I) and Au(I) is exemplified by the characteristic downfield shifts in ^1^H NMR peaks as well as peak integration agreements (Supplementary Figures 2, 3A). In addition, the same peak splitting patterns between the ligand and the complex/hexagon were expected due to the C_2v_ symmetry of both Ag**L1**_2_ and Au**L1**_2_ complexes and the D_6h_ symmetry of all {Ag**L**}_6_ hexagonal metallacycles.

For the Au(I) hexagons, ligand dissociation occurs when MeCN, DMSO, and acetone were used as solvents. These hexagons are insoluble in all other solvents except for nitromethane, in which we were able to observe intact Au(I) hexagons. Figure 3 summarizes the ^1^H NMR spectra of {Au**L2**}_6_, {Au**L3**}_6_, and {Au**L4**}_6_. The full spectra of each Au(I) hexagon are shown in Supplementary Figures 3B–D. Similar to the Ag(I) hexagons, the downfield shifts of all ^1^H NMR peaks, the peak integration agreements, and the similarity in peak splitting patterns indicate the formation of pure Au(I) hexagonal metallacycles. Although the solubility of these species is limited, our identification of NMR-suitable solvents rules out the formation of polymeric species as such coordination polymers are fully insoluble. ESI-MS (see below) further confirms the stoichiometries of self-assembly.

### Formation of Ag(I) and Au(I) Complexes and Hexagons in Acetone

All of these complexes were synthesized in pure CHCl_3_ or in CHCl_3_ mixtures with MeOH. MeCN and DMSO competitively coordinate with the Ag(I) and Au(I) resulting in ligand dissociation. Acetone was a suitable solvent for the building blocks, as well as the final assemblies, enabling the study of complex formation. Using ^1^H NMR methods, we initially acquired a series of spectra for a solution containing 1:1 equivalents of Ag:**L** in acetone upon mixing and after 24 h (Supplementary Figure 4). We observed that the thermodynamic products formed immediately upon mixing with no significant changes observed after 24 h. This suggests a fast kinetics with a large formation constant for all Ag(I) complex and hexagons in acetone.

Similarly, we wanted to study the solution formation of the Au(I) complex and hexagons. In this case, we prepared solutions containing 1:1, 2:1, and 3:1 equivalents of Au:**L** in acetone to ensure complete ligand coordination. The acquired ^1^H NMR spectra at 0 (upon mixing), 6, 24, and 48 h were summarized in Supplementary Figures 5–7 for 1:1, 2:1, and 3:1 solutions, respectively. It was observed that the Au**L1**_2_ complex self-assembles within 6 h then partially dissociates thereafter. In the case of both {Au**L2**}_6_ and {Au**L3**}_6_, an equilibrium was established immediately upon mixing that stays up to at least 48 h. Products of {Au**L4**}_6_ hexagon assembly crash out of solution immediately upon mixing such that we only observe uncoordinated **L4** and partially dissolved products. In all cases, the initial coordination is fast; however, quantitative analyses of the NMR peaks is not straightforward using a one-step metal-ligand binding model. There are reports on the mechanism and reducing ability of acetone to form Au nanoparticles from Au(III) and Au(I) (Marin et al., 2008). Thus, we think that there is a possibility of either Au(I) slowly being released as Au(0) from the hexagonal metallacycle into the acetone solution or acetone competitively coordinating to Au(I) to form other products.

### FT-ICR MS of Ag(I) and Au(I) Complexes and Hexagons

High-resolution mass spectrometry (HRMS) was used to probe the stoichiometry of our self-assemblies. We used Fourier transform-ion cyclotron mass spectrometry (FT-ICR MS) with electrospray ionization (ESI) for soluble complexes and hexagons, while laser desorption ionization (LDI) was used for hexagons that are unstable in acetone. ESI, as a soft ionization technique, is advantageous and popularly used in detection of intact coordination complexes and cages with high molar masses (Vikse and Scott McIndoe, 2018). Typical of soft ionization methods, molecules are ionized by either loss of ions or association with low-valent ions. In the case of our Ag(I) and Au(I) complexes and hexagons, loss of counter-ions results in multiply-charged compounds that are easily detectable by HR-MS at lower m/z. In cases wherein the hexagon is not stable in acetone, we opted to use matrix-free LDI, which uses high-energy lasers to ionize samples in the solid state. LDI is not popularly used in coordination chemistry due to formation of singly-charged fragment ions (Mandal et al., 2019). We, nonetheless, successfully identified intact hexagons in their solid state.

**Figure 4:**
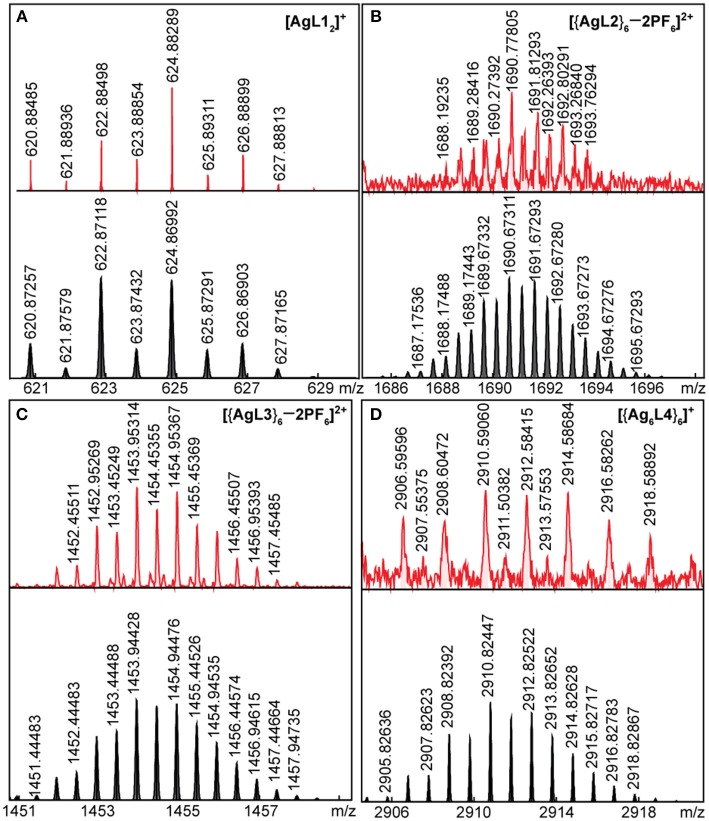
ESI-FT-ICR MS spectrograms of intact **(A)** Ag**L1**_2_, **(B)** {Ag**L2**}_6_, **(C)** {Ag**L3**}_6_, and **(D)** LDI-FT-ICR MS spectrum of intact {Ag**L4**}_6_ (top = experimental, bottom = simulated).

**Figure 5:**
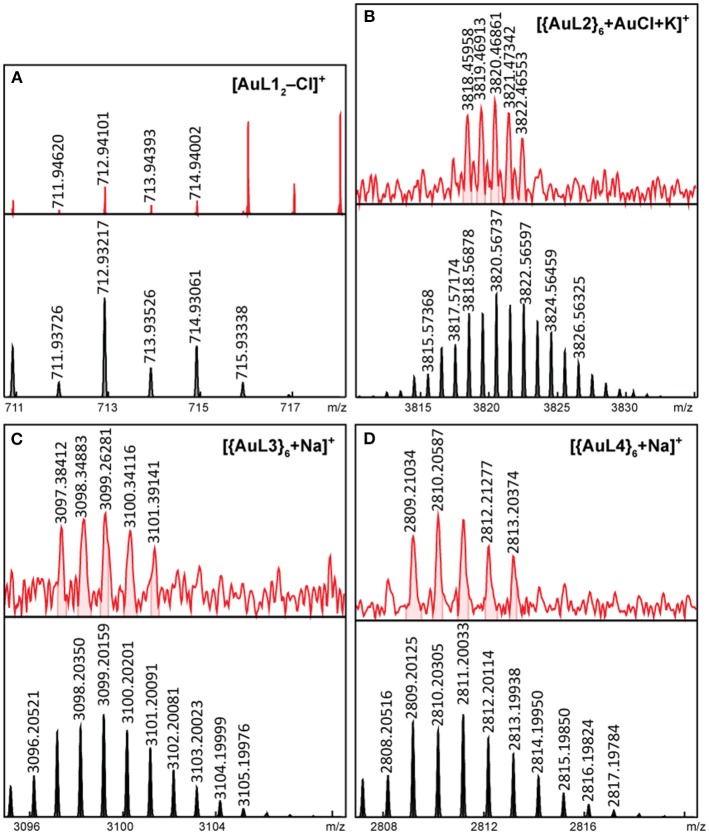
ESI-FT-ICR MS spectrogram of intact **(A)** Au**L1**_2_ and LDI-FT-ICR MS spectrograms of intact **(B)** {Au**L2**}_6_, **(C)** {Au**L3**}_6_, and **(D)** {Au**L4**}_6_ (top = experimental, bottom = simulated).

The mass spectrograms for Ag(I) complex and hexagons and for Au(I) complex and hexagons are shown in Figures 4, 5, respectively. For the Ag(I) complex and hexagons, we were able to detect the intact [Ag**L1**_2_]^+^ complex (Figure 4A) as well as [{Ag**L2**}_6_ – 2PF_6_]^2+^ hexagon (Figure 4B) and [{Ag**L3**}_6_ – 2PF_6_]^2+^ hexagon (Figure 4C) by ESI-FT-ICR MS. A loss of one ${\text{PF}}_{6}^{-}$ counter-ion was observed for the complex and two ${\text{PF}}_{6}^{-}$ were observed for both hexagons. [{Ag**L4**}_6_]^+^ hexagon (Figure 4D), which has low solubility in acetone, was detected by LDI-FT-ICR MS.

For the Au(I) complex and hexagons, we were able to detect the intact [Au**L1**_2_]^+^ complex (Figure 5A) with a loss of Cl^−^ counter-ion by ESI-FT-ICR MS. The Au(I) hexagons, which are unstable is acetone were detected by LDI-FT-ICR MS. We were able to detect singly-charged intact [{Au**L2**}_6_+AuCl+K]^+^ hexagon (Figure 5B), [{Au**L3**}_6_+Na]^+^ hexagon (Figure 5C), and [{Au**L4**}_6_+Na]^+^ hexagon (Figure 5D). Each of these hexagons have six chloride counter-ions. We also observed {Au**L**}_6_ fragments (Supplementary Figures 12–15) with chloride counter-ions by ESI-FT-ICR MS, which further confirms the presence of chloride counter-ions in the Au(I) hexagons.

In all cases, we were able to confirm the presence of intact complexes and cages from the agreement of molecular ion m/z and isotopic distribution pattern between the simulated and experimental spectrograms. The presence of complex and hexagon fragmentation (Supplementary Figures 8–11 for Ag(I) and Supplementary Figures 12–15 for Au(I) complexes and hexagons) in the mass spectra further confirmed the structure and counter-ions of the linear complex and hexagonal self-assemblies since these fragments were not detected in the NMR studies.

### Structures of Ag(I) and Au(I) Complexes and Hexagons

**Figure 6:**
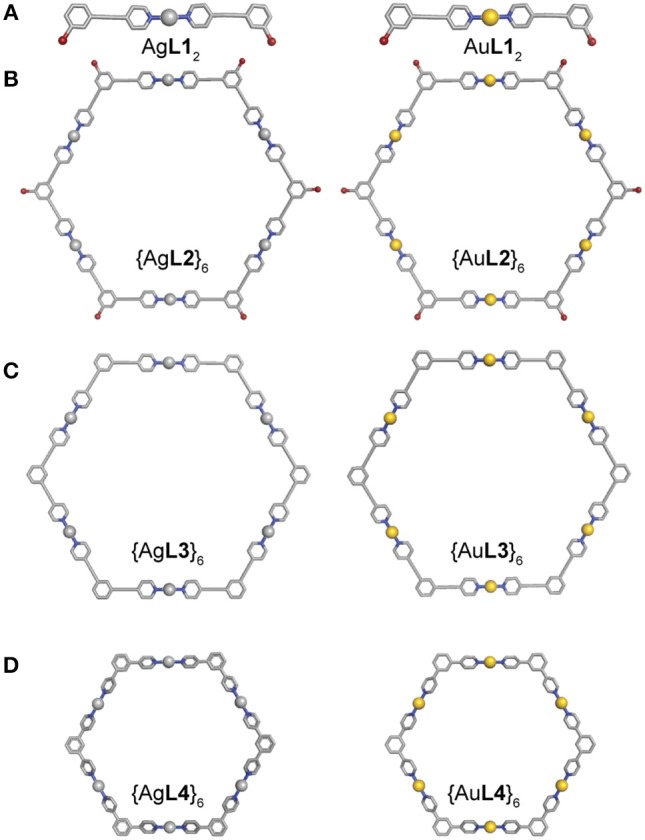
Molecular models from crystal structure of **(A)** Ag**L1**_2_ (CCDC 1879931) and Universal Force Field (UFF) optimized structures of Au**L1**_2_, **(B)** {Ag**L2**}_6_ and {Au**L2**}_6_, **(C)** {Ag**L3**}_6_ and {Au**L3**}_6_, and **(D)** {Ag**L4**}_6_ and {Au**L4**}_6_ (C = gray, N = blue, Br = maroon, Ag = silver, and Au = gold).

Single-crystal X-ray diffraction was attempted to further elucidate the structures of the Ag(I) and Au(I) complexes and hexagons. Slow evaporation of acetone solutions resulted in very fine needle-like crystals. Only crystals of Ag**L1**_2_ gave a tractable data set. The asymmetric unit contains two formula units (Z′ = 2) held in a cofacial conformation as well as a single chloroform molecule. Each Ag(I) center possess a coordination number of 2 with near ideal 180° geometries (Figure 6A and Supplementary Figure 16 and Table S1). The PF_6_- counter ion is outer sphere with a closest contact of approximately 3 Å. It is apparent that non-covalent interactions are a strong driving force in the resulting crystal structure of this compound. An apparent argentophilic Ag(I)—Ag(I) interaction leads to the interesting cofacial conformation (~3.5 Å). Furthermore, interaction between the electron-rich Br atoms and the H atoms (average separation 3.60(9) Å) of the corresponding formula unit further contribute to the observed structure.

Molecular mechanics geometry optimizations were carried out on the Au(I) complex and the Ag(I) and Au(I) hexagons (Figures 6B–D). These structures were optimized using Universal Force Field (UFF) as implemented in ArgusLab. All hexagons formed from **L2** and **L3** preserved ligand planarity. In contrast, the hexagons from **L4** have pyridyl groups that are rotated by 40° from the central phenyl due to the steric strain of the protons on neighboring aromatic rings.

### Solid-State Light Stability of Ag(I) and Au(I) Complexes and Hexagons

**Figure 7:**
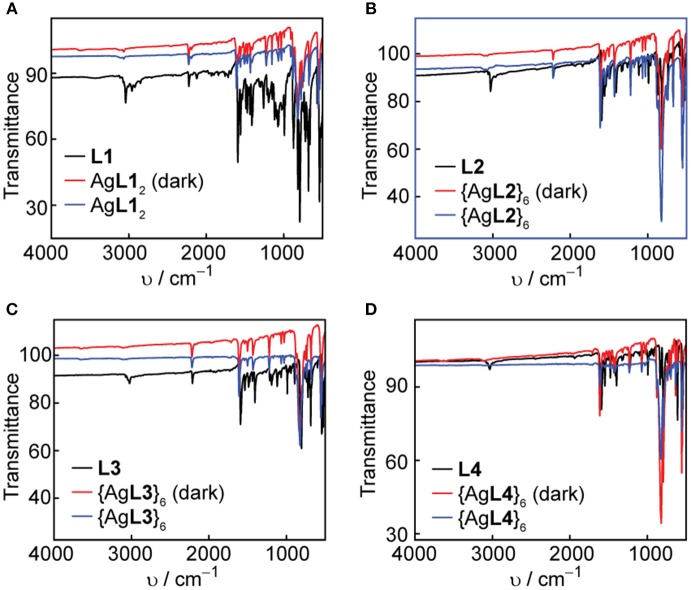
Comparison of FT-IR spectra in the dark and under room light conditions for **(A) L1** and Ag**L1**_2_, **(B) L2** and {Ag**L2**}_6_, **(C) L3** and {Ag**L3**}_6_, and **(D) L4** and {Ag**L4**}_6_.

**Figure 8:**
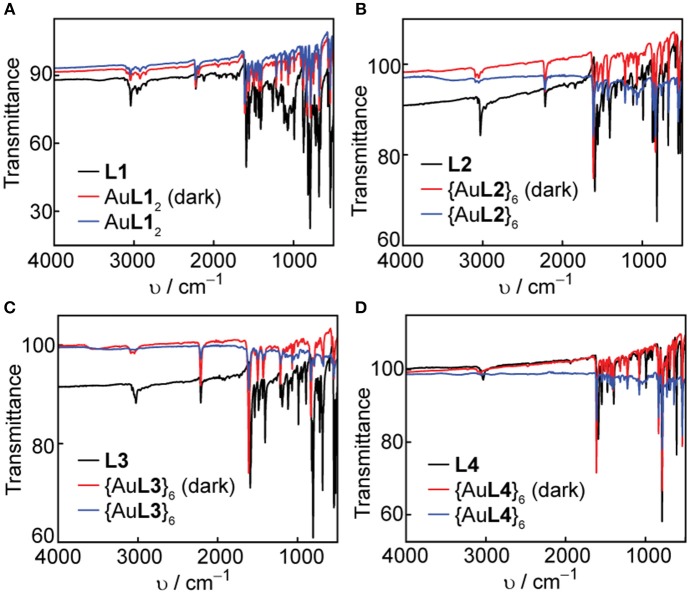
Comparison of FT-IR spectra in the dark and under room light conditions for **(A) L1** and Au**L1**_2_, **(B) L2** and {Au**L2**}_6_, **(C) L3** and {Au**L3**}_6_, and **(D) L4** and {Au**L4**}_6_.

Solid-state IR spectroscopy is another useful tool to confirm the coordination of the pyridyl ligands to Ag(I) and Au(I) metal acceptors and study the solid-state photostability of the complexes and hexagons. We acquired FT-IR spectra of the ligands, complexes and hexagons as synthesized. After a week of storing the complexes and hexagons under room light at ambient conditions, we re-acquired the FT-IR spectra of each compound. The ligand IR spectra are shown in both Figures 7, 8, where the characteristic aromatic -CH stretching at 3,000–3,100 cm^−1^, aromatic -CC- stretching at 1,400–1,600 cm^−1^, and aromatic -CN- stretching at 1,600 cm^−1^ are present in all ligands (Katritzky and Hands, 1958; Schrader, 2007). The characteristic alkyne -CC- stretching at 2,100 cm^−1^ are also present in **L1**, **L2**, and **L3**.

In all cases, there is a slight shift to higher wavenumbers upon Ag(I) or Au(I) coordination, most notably for the -CN- stretching peaks at 1,600 cm^−1^. We observe no change in both the functional group and the fingerprint regions after exposure of the complexes and hexagons to room light for one week, which confirms the improved photo-stability of Ag(I) and Au(I) upon coordination into both linear complex and hexagonal metallacycle.

### Photophysical Properties of Ag(I) and Au(I) Complexes and Hexagons

The photophysical properties of these self-assembled complexes and hexagons were studied using steady-state and time-resolved methods. Studies of Ag(I) (Zhou et al., 2006; Laye, 2007; Yeşilel et al., 2012; Mei et al., 2015a; Jenkins and Assefa, 2017) and Au(I) (Lin et al., 2008; Langdon-Jones and Pope, 2014; Shakirova et al., 2017) complexes and metallacycles with N-donor heterocyclic ligands demonstrate interesting luminescent properties, which assigned to metal-to-ligand charge transfers (MLCT), ligand-based charge transfers, or metal-perturbed ligand-based charge transfers in part due to argentophilic or aurophilic interactions.

**Figure 9:**
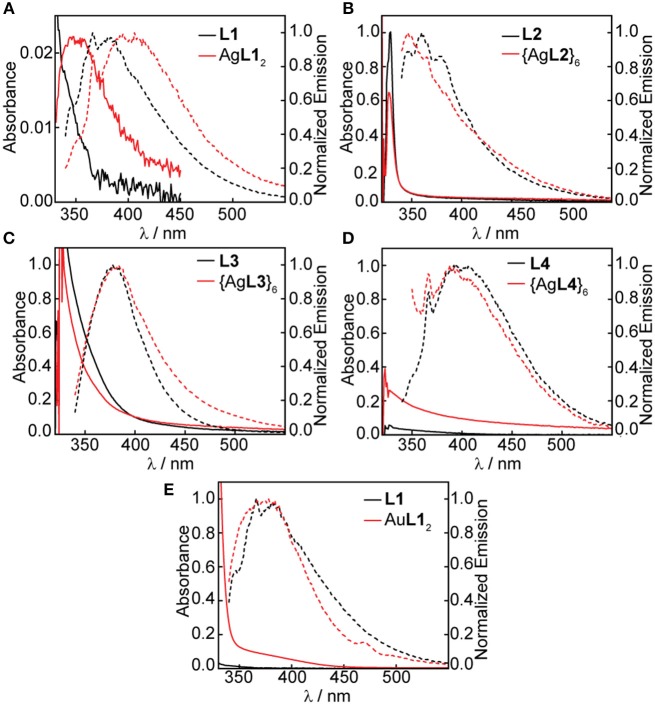
UV-vis absorbance and emission spectra in acetone of **(A)** 0.10 mM **L1** and saturated Ag**L1**_2_, **(B)** 0.10 mM **L2** and saturated {Ag**L2**}_6_, **(C)** 0.10 mM **L3** and saturated {Ag**L3**}_6_, and **(D)** 1.0 mM **L4** and saturated {Ag**L4**}_6_, **(E)** 0.10 mM **L1** and 0.10 mM Ag**L1**_2_.

We studied both the solution- and solid-state photoluminescence of all Ag(I) and Au(I) complexes and hexagons; however, the acetone instability of some compounds limited our solution-state studies to the photoluminescence of Ag(I) complexes and hexagons and the mononuclear Au(I) complex in acetone. The solution-state absorption and emission of the free ligands, Ag(I) and Au(I) complexes, and Ag(I) hexagons are summarized in Figure 9. Several UV-vis absorption features were masked by the UV-cutoff of acetone. Nonetheless, the high energy band due to π → π^*^ ligand-centered transitions that is typical of aromatic and alkynyl-containing ligands (Shakirova et al., 2017) were prominent in **L1**, **L2**, and **L3** and less so in **L4** due to the absence of ethynyl spacer in this ligand. We see a slight red-shift in the absorption of Ag**L1**_2_ (Figure 9A) but not in Au**L1**_2_ (Figure 9E) or any of the Ag(I) hexagons (Figures 9B–D) suggesting ligand-centered transitions for the Au(I) complex and Ag(I) hexagons and metal-perturbed ligand-centered transition for the Ag(I) complex, which is supported by the presence of argentophilic interactions in the crystal structure (Figure 6).

Table 1 shows the summary of all measured quantum yields (Φ, Supplementary Figures 17–22) and lifetimes (τ, Supplementary Figures 23–25) and all calculated radiative (*k*_*r*_) and non-radiative (*k*_*nr*_) decay rate constants. At 330 nm excitation, fluorescence emission bands centered at 375 nm (Figures 9A–C) were observed for **L1**, **L2**, and **L3** in acetone at 3.82, 0.800, and 7.76% quantum yields and 0.11, 0.12, 0.49 ns lifetimes, respectively (Table 1). These ligands have ethynyl spacers that connect the central phenyl group to the pyridyl group/s, which helps rigidify the structure and enhance emission. The highest quantum yield and longest lifetime was observed in **L3**, which is not affected by heavy-atom effects, unlike **L1** and **L2** wherein bromide was present. In addition, the four-fold increase in *k*_*nr*_ for **L1** (8.91 × 10^9^ s^−1^) and **L2** (8.00 × 10^9^ s^−1^) as compared to **L3** (1.89 × 10^9^ s^−1^), further supports the enhancement of inter-system crossing (ISC) for the bromine-containing ligands. On the other hand, a weak fluorescence band centered at 400 nm (Figure 9D) was observed for **L4** but has a quantum yield and lifetime below the limit of our instrumental detection. By placing an upper bound on quantum yield and lifetime of **L4** based on instrumental detection limits, we estimated an upper limit to *k*_*r*_ and a lower limit to *k*_*nr*_ as shown in Table 1. The absence of the ethynyl spacer in **L4** introduces rotational freedom that enhances internal conversion resulting in very weak emission of the ligand.

**Table 1:** Solution- and solid-state quantum yields and lifetimes of ligands, Ag and Au complexes and hexagons.

**Compound**	**Φ_solid-state_, %**	**Φ_solution-state_,^a^ %**	**τ, ns**	***k_*r*_*, s^−1^**	***k_*nr*_*, s^−1^**
L1	0.480	3.82	0.11	3.54 x 10^8^	8.91 x 10^9^
L2	0.180	0.800	0.12	6.45 x 10^7^	8.00 x 10^9^
L3	1.190	7.76	0.49	1.59 x 10^8^	1.89 x 10^9^
L4	0.010	~0.01^*^	0.10^**^	<1.00 x 10^6^	>1.00 x 10^10^
AgL1_2_	0.200	2.32	0.10^**^	>2.32 x 10^8^	>9.77 x 10^9^
{AgL2}_6_	0.340	0.300	0.10^**^	>3.00 x 10^7^	>9.97 x 10^9^
{AgL3}_6_	0.130	4.70	0.17	2.83 x 10^8^	5.74 x 10^9^
{AgL4}_6_	0.510	~0.01^*^	0.10^**^	<1.00 x 10^6^	>1.00 x 10^10^
AuL1_2_	0.06	1.12	0.11	1.04 x 10^8^	9.19 x 10^9^
{AuL2}_6_	0.170	^***^	^***^	^***^	^***^
{AuL3}_6_	0.010	^***^	^***^	^***^	^***^
{AuL4}_6_	0.110	^***^	^***^	^***^	^***^

a *solution-state quantum yield all measured in acetone*.

* *measured QY is below the instrumental limit of detection of 0.01%*.

** *measured lifetime is below the instrumental limit of detection of 0.10 ns*.

*** *QY not measured due to instability in acetone*.

Similar to the absorption profiles, we also observe a red-shift in the emission band of Ag**L1**_2_ centered at 400 nm (Figure 9A) but not in Au**L1**_2_ (375 nm, Figure 9E) or any of the Ag(I) hexagons, {Ag**L2**}_6_ (375 nm, Figure 9B), {Ag**L3**}_6_ (375 nm, Figure 9C), and {Ag**L4**}_6_ (400 nm, Figure 9D). These emission profiles are similar to that of the ligand with significant reduction in quantum yields and lifetimes upon ligand coordination to Ag(I) and Au(I) suggesting ligand-centered singlet emissions with some perturbations from Ag(I) argentophilic interactions for the Ag(I) complex. Although not as common, fluorescent transition metal complexes are known (Chia and Tay, 2014). The lack of triplet emissions in these complexes can be attributed to either lack of metal-ligand interaction resulting in small metal contribution at the excited state or larger rate constant for fluorescence compared to that of ISC, which is reasonable given the fluorescence emission of the free ligand. Based on our decay rate constant calculations for the Ag(I) and Au(I) complexes and Ag(I) hexagons, *k*_*nr*_ is always higher than *k*_*r*_ which implies poor metal-ligand interaction as the main reason for lack of phosphorescence in these compounds.

We also reported the solid-state quantum yields of all ligands, Ag(I) and Au(I) complexes, and hexagons in Table 1. Similar to the solution-state quantum yields, the highest and lowest quantum yields are observed for **L3** are **L4**, respectively. Upon ligand coordination to either Ag(I) and Au(I), we see a general decrease in quantum yields except for {Ag**L2**}_6_, {Ag**L4**}_6_, and {Ag**L4**}_6_. These quantum yield enhancement may be due to aggregation induced emission (Fan et al., 2015; Mei et al., 2015b), particularly in metallacycles containing **L4**, which in itself is weakly emissive, wherein non-radiative pathways are attenuated when intramolecular motions are reduced.

## Conclusion

Silver(I) and gold(I) mononuclear complexes and hexagonal rings were self-assembled from AgPF_6_ and Au(tht)Cl precursors and mono- and bidentate pyridyl ligands. These coordination resulting in self-assembly and complex formation was evidenced by the downfield ^1^H NMR peak shifts of the ligand pyridyl H peaks and FT-ICR MS peaks with isotopic distributions and mass-to-charge ratios consistent with intact [6 + 6] cores that ionized by the loss of counterions.

Longer-term photostability of all complexes and hexagons in the solid-state were confirmed by FT-IR. Significant peak shifts to higher wavenumbers were observed upon coordination of the ligands to either Ag(I) or Au(I). No changes to the spectra were observed after storing the compounds at ambient conditions under room light for one week.

Ligand-centered fluorescence emission was observed for many of these complexes. In both the solid- and solution-state, **L3** and **L4** were the most and least emissive ligands, respectively. In the solid-state, all complexes and hexagons have diminished emission except for {Ag**L2**}_6_, {Ag**L4**}_6_, and {Au**L4**}_6_ wherein aggregation-induced emission was observed. In the solution-state, all ligands and hexagons exhibited ligand-centered singlet emissions while Ag**L1**_2_ and Au**L1**_2_ both exhibited metal-perturbed ligand-centered singlet emissions.

This work establishes that Ag(I) and Au(I) centers are effective linear nodes for self-assembly reactions with dipyridyl ligands and that the resultant materials stabilize these ions with respect to oxidation and photodecomposition. Due to the modular nature of coordination-driven self-assembly, efforts are ongoing to exploit ligand-tuning to incorporate functional groups that will enhance metallacycle solubility to improve their processability for incorporation into mixed-matrix materials.

## Data Availability

The dataset Ag**L1**_2_ (CCDC No. 1879931) for this study can be found in the Cambridge Crystallographic Data Centre https://www.ccdc.cam.ac.uk/solutions/csd-system/components/csd/.

## Author Contributions

CF designed and carried out the experiments and wrote the manuscript. SK assisted in ligand synthesis. AF carried out the mass spec characterization. TC designed the project and wrote the manuscript.

### Conflict of Interest Statement

The authors declare that the research was conducted in the absence of any commercial or financial relationships that could be construed as a potential conflict of interest.
