# Supplementary material for: Coordination-Driven Self-Assembly of Silver(I) and Gold(I) Rings: Synthesis, Characterization, and Photophysical Studies
Source: Front Chem. 2019 Aug 13;7:567. doi: 10.3389/fchem.2019.00567 (PMC6705220; doi:10.3389/fchem.2019.00567)
Supplement: Supplementary file 1 [file Table_1.DOCX]

Supplementary Material

Table of Contents

[1 Experimental 2](#_Toc2075929)

[1.1 Synthetic Methods 2](#_Toc2075930)

[1.1.1 Synthesis of 1-bromo-3(4-ethynylpyridyl)benzene (L1) 2](#_Toc2075931)

[1.1.2 Synthesis of 5-bromo-1,3-bis(4-ethynylpyridyl)benzene (L2) 2](#_Toc2075932)

[1.1.3 Synthesis of 1,3-bis(4-ethynylpyridyl)benzene (L3) 2](#_Toc2075933)

[1.1.4 Synthesis of 1,3-bis(4-pyridyl)benzene (L4) 3](#_Toc2075934)

[1.1.5 Synthesis of tetrahydrothiophenegold(I) chloride (Au(tht)Cl) 3](#_Toc2075935)

[1.2 Quantum yield (Φ) determination 3](#_Toc2075936)

[1.3 Time-correlated Single Photon Counting (TCSPC) Fluorescence Lifetime (τ) 4](#_Toc2075937)

[1.4 Radiative (k_r_) and non-radiative (k_nr_) decay constant calculations 4](#_Toc2075938)

[2 ^1^H NMR Characterization 5](#_Toc2075939)

[3 K_f_ Determination using ^1^H NMR 8](#_Toc2075940)

[4 FT-ICR MS Characterization 11](#_Toc2075941)

[5 Crystal Structure Details for AgL1_2_ 19](#_Toc2075942)

[6 Solid- and Solution-State Φ Determination 21](#_Toc2075943)

[7 TCSPC Lifetime Determination 27](#_Toc2075944)

[8 References 29](#_Toc2075945)

# Experimental

## Synthetic Methods

### Synthesis of 1-bromo-3(4-ethynylpyridyl)benzene (L1)

**L1** was synthesized using a modified literature procedure.(Lo et al., 2009) A 100-mL Schlenk flask was charged with 0.431 g (3.09 mmol) 4-ethynylpyridine hydrochloride, 0.030 g (0.16 mmol) CuI, and Teflon-coated magnetic spin bar then transferred into a glovebox under N_2_ atmosphere. Inside the glovebox, 0.083 g (0.072 mmol) Pd(PPh_3_)_4_, 3.0 mL dry NEt_3_, and 3.0 mL dry THF were added to the flask. After 5 min of stirring, the flask was sealed and transferred into a Schlenk line. Then 0.248 g (1.05 mmol) 1,3-dibromobenzene was added into the flask under positive N_2_ pressure. The mixture was refluxed under N_2_ at 60°C for 18 h. After the reaction, the mixture was concentrated in vacuo and 50.0 mL DCM was added. The resulting solution was washed with 50.0 mL brine (3x), dried with 2.0 g Na_2_SO_4_, filtered, and concentrated. The product was purified in a silica gel column using 1:1 DCM:EtOAc, re-dissoved in 1:1 DCM:hexane, filtered, and concentrated in vacuo. Pure product was isolated as a yellow powder (0.106 g, 39% yield). ^1^H NMR (400 MHz, acetone-d_6_, 25°C): δ (ppm) = 8.63 (d, ^3^J = 6.0 Hz, 2H, pyridyl H_α_), 7.78 (s, 1H, phenyl H), 7.65 (d, ^3^J = 7.1 Hz, 1H, phenyl H), 7.60 (d, ^3^J = 7.8 Hz, 1H, phenyl H), 7.49 (d, ^3^J = 6.0 Hz, 2H, pyridyl H_β_), 7.42 (t, ^3^J = 7.9 Hz, 1H, phenyl H). FT-IR (ATR, cm^–1^): 3045 (w), 2235 (w), 1597 (m), 1550 (m), 1493 (w), 1468 (w), 1436 (w), 1411 (w), 1257 (w), 1194 (w), 1153 (w), 1118 (w), 1093 (w), 1074 (w), 1027 (w), 992 (w), 873 (m), 816 (s), 790 (s), 746 (w), 737 (m), 718 (m), 696 (m), 677 (s), 661 (m), 570 (w), 538 (s), 529 (s).

### Synthesis of 5-bromo-1,3-bis(4-ethynylpyridyl)benzene (L2)

**L2** was synthesized using a modified literature procedure.(Wiesler et al., 2001) A 100-mL Schlenk flask was charged with 0.500 g (1.60 mmol) 1,3,5-tribromobenzene, 0.444 g (3.18 mmol) 4-ethynylpyridine hydrochloride, 0.003 g (0.016 mmol) CuI, and Teflon-coated magnetic spin bar then transferred into a glovebox under N_2_ atmosphere. Inside the glovebox, 0.218 g (0.31 mmol) Pd(PPh_3_)_2_Cl_2_, 4.0 mL dry NEt_3_, and 5.0 mL dry toluene were added to the flask. After 5 min of stirring, the flask was sealed and transferred into a Schlenk line. The mixture was refluxed under N_2_ at 90°C for 48 h. After the reaction, the mixture was concentrated and 50.0 mL DCM was added. The resulting solution was washed with 50.0 mL brine (3x), dried with 2.0 g Na_2_SO_4_, filtered, and concentrated. The product was purified in a silica gel column using 1:1 DCM:EtOAc. Pure product was isolated as a white powder (0.131 g, 23% yield). ^1^H NMR (400 MHz, Acetone-d_6_, 25°C): δ (ppm) = 8.66 (d, ^3^J = 6.0 Hz, 4H, pyridyl H_α_), 7.87 (s, 2H, phenyl H), 7.82 (s, 1H, phenyl H), 7.53 (d, ^3^J = 6.0 Hz, 4H, pyridyl H_β_). FT-IR (ATR, cm^–1^): 3029 (w), 2212 (w), 1593 (m), 1556 (m), 1531 (w), 1481 (w), 1404 (m), 1338 (w), 1322 (w), 1260 (w), 1210 (w), 1116(w), 989 (w), 960 (w), 866 (m), 817(s), 730 (w), 677 (m), 583 (w), 550 (m), 521 (m).

### Synthesis of 1,3-bis(4-ethynylpyridyl)benzene (L3)

**L3** was synthesized using a modified literature procedure.(Wiesler et al., 2001) A 250-mL Schlenk flask was charged with 5.92 g (42.4 mmol) 4-ethynylpyridine hydrochloride, 0.028 g (0.15 mmol) CuI, and Teflon-coated magnetic spin bar then transferred into a glovebox under N_2_ atmosphere. Inside the glovebox, 1.52 g (2.17 mmol) Pd(PPh_3_)_2_Cl_2_, 30.0 mL dry NEt_3_, and 30.0 mL dry toluene were added to the flask. After 5 min of stirring, the flask was sealed and transferred into a Schlenk line. Then 4.000 g (16.96 mmol) 1,3-dibromobenzene was added into the flask under positive N_2_ pressure. The mixture was refluxed under N_2_ at 90°C for 48 h. The product was purified in a silica gel column using 95:5 DCM:MeOH, re-dissolved in 50.0 mL 1:1 DCM:hexane, filtered, and concentrated in vacuo. Pure product was isolated as a yellow powder (2.973 g, 63% yield). ^1^H NMR (400 MHz, Acetone-d_6_, 25°C): δ (ppm) = 8.65 (d, ^3^J = 6.0 Hz, 4H, pyridyl H_α_), 7.83 (s, 1H, phenyl H), 7.69 (d, ^3^J = 8.9 Hz, 2H, phenyl H), 7.59 – 7.53 (m, 1H, phenyl H), 7.51 (d, ^3^J = 6.0 Hz, 4H, pyridyl H_β_). FT-IR (ATR, cm^–1^): 3029 (w), 2210 (w), 1596 (m), 1534 (w), 1482 (w), 1436 (w), 1409 (m), 1210 (w), 1191 (w), 1118 (w), 1069 (w), 986 (m), 941 (w), 895 (w), 824 (m), 803 (m), 754 (w), 738 (w), 720 (m), 686 (m), 594 (w), 542 (m), 521 (m).

### Synthesis of 1,3-bis(4-pyridyl)benzene (L4)

**L4** was prepared using a modified literature procedure.(Sun et al., 2015) A 100-mL Schlenk flask was charged with 0.573 g (4.66 mmol) pyB(OH)_2_, 2.900 g (20.98 mmol) K_2_CO_3_, and Teflon-coated magnetic spin bar then transferred into a glovebox under N_2_ atmosphere. Inside the glovebox, 0.245 g (0.21 mmol) Pd(PPh_3_)_4_ was added to the flask. The flask was sealed and transferred into a Schlenk line. In a separate 100-mL Schlenk flask, 0.500 g (2.12 mmol) 1,3-dibromobenzene, 18.0 mL filtered and dried DMF, and 0.42 mL water were added. This mixture was freeze-pump-thawed three times then cannula-transferred into the reaction flask. The reaction mixture was refluxed under N_2_ at 100°C for 48 h. After the reaction, the mixture was filtered through a Celite plug and extracted with 100.0 mL chloroform and 50.0 mL brine (3x). The product was purified in a silica gel column using 96:4 DCM:MeOH. The collected product was concentrated in vacuo and washed with 10-mL portions of diethyl ether and petroleum ether. Pure product was isolated as a yellow powder (0.359 g, 73% yield). ^1^H NMR (400 MHz, Acetone-d_6_, 25°C): δ (ppm) = 8.68 (d, ^3^J = 6.1 Hz, 4H, pyridyl H_α_), 8.17 (s, 1H, phenyl H), 7.89 (d, ^3^J = 9.5 Hz, 2H, phenyl H), 7.78 (d, ^3^J = 6.1 Hz, 4H, pyridyl H_β_).), 7.70 (t, ^3^J = 7.8 Hz, 1H, phenyl H). FT-IR (ATR, cm^–1^): 3037 (w), 1590 (m), 1549 (w), 1476 (w), 1438 (w), 1391 (w), 1324 (w), 1224 (w), 1187 (w), 1096 (w), 1073 (w), 994 (w), 962 (w), 932 (w), 912 (w), 834 (m), 825 (m), 792 (s), 737 (w), 702 (w), 667 (w), 640 (w), 605 (m), 533 (m).

### Synthesis of tetrahydrothiophenegold(I) chloride (Au(tht)Cl)

Au(tht)Cl was prepared using a modified literature procedure.(Uson et al., 2007) In a 20-mL scintillation vial charged with a Teflon-coated magnetic spin bar, 0.547 g (1.39 mmol) HAuCl_4_⋅3H_2_O, 1.0 mL water, and 5.0 mL EtOH were added. Then 0.258 mL (2.92 mmol) tht was added dropwise. The mixture was stirred for 15 min, then filtered. The powder collected was washed with 10-mL portions of EtOH. The isolated white powder product (0.446, 100% yield) was stored in the dark inside a desiccator (Drierite) until use.

## Quantum yield (Φ) determination

Solid-state quantum yields (*Φ*) were measured for all Ag(I) and Au(I) complexes and hexagons while solution-state quantum yields were measured for 0.10 mM solutions of Ag(I) and Au(I) complexes and saturated solutions of Ag(I) hexagons in acetone. The optimized parameters used for both sample and blank excitation range emission spectra acquisitions are 330 nm excitation wavelength, 320–340 nm emission wavelengths, 1 nm and 1.5 nm excitation and emission slit widths, respectively, 0.50 OD neutral density filter on the entrance slit, and 4-averaged scans of 1-s integrations. Similar parameters was used for the emission range emission spectra acquisition except for the emission range of 340–360 nm, no neutral density filter used, and 4-averaged scans of 4-s integrations.

Quantum yields were calculated using FluorEssence v3.5 PLQY software that employs the following equation:

$$\Phi=\frac{E_{c}-E_{a}}{L_{a}-L_{c}}\times N$$

where *E_c_* = integrated area under the curve of sample emission at emission range, *E_a_* = integrated area under the curve of blank emission at emission range, *L_a_* = integrated area under the curve of sample emission at excitation range, *L_c_* = integrated area under the curve of sample emission at excitation range, and *N* = 12.7 (correction factor based on differences in integration time and neutral density filter used for excitation and emission ranges). The instrumental limit of detection is 0.01%, therefore all calculated quantum yields below this limit was estimated as <0.01%.

**Equation 1**

## Time-correlated Single Photon Counting (TCSPC) Fluorescence Lifetime (τ)

The lifetimes (*τ*) of 0.10 mM solutions of Ag(I) and Au(I) complexes and saturated solutions of Ag(I) hexagons in acetone were measured using TCSPC. Prompt and decay traces were acquired at 100 ns time range, 350 nm excitation wavelength, 375 nm emission wavelength, 950 V operating voltage, and 10,000 counts total number of decay points. Lifetimes were determined using DAS6 v6.8 Analysis software to fit the collected traces to a mono-, bi-, and tri-exponential decay with the lowest χ^2^ considered as the best-fit model. The instrumental limit of detection is 0.1 ns therefore all calculated lifetimes below the limit were estimated as >0.1 ns.

## Radiative (k_r_) and non-radiative (k_nr_) decay constant calculations

The rate constants of the radiative (*k_r_*) and non-radiative (*k_nr_*) pathways for the emission decay of all solutions with measurable quantum yields and lifetimes were calculated using the following equation:

$$\Phi=\frac{k_{r}}{k_{r}+k_{nr}}$$

where the inverse of estimated lifetime, 1/*τ* = *k_r_ + k_nr_*. For compounds with *Φ* and *τ* below the instrumental limit of detection, we report the estimated upper-bounds of *k_r_* and lower-bounds of *k_nr_*. For compounds with *τ* below the instrumental limit of detection, we report the estimated lower-bounds of both *k_r_* and *k_nr_*.

**Equation 2**

# ^1^H NMR Characterization


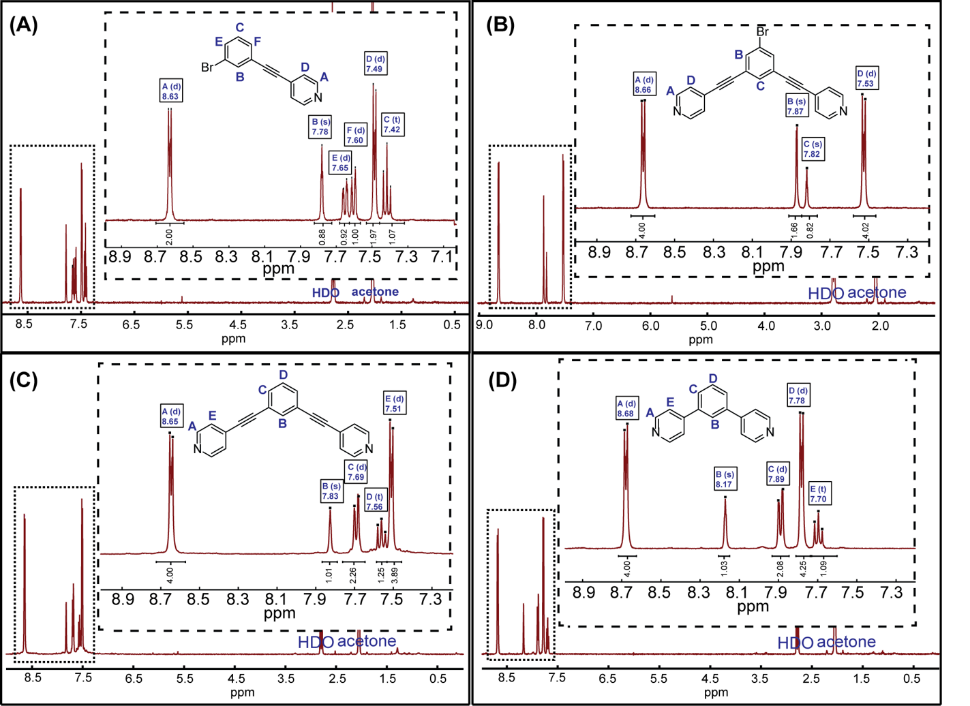


**Supplementary Figure 1**. ^1^H NMR spectra of ligands **(A)** **L1**, **(B)** **L2**, **(C)** **L3**, and **(D)** **L4** in acetone-d_6_


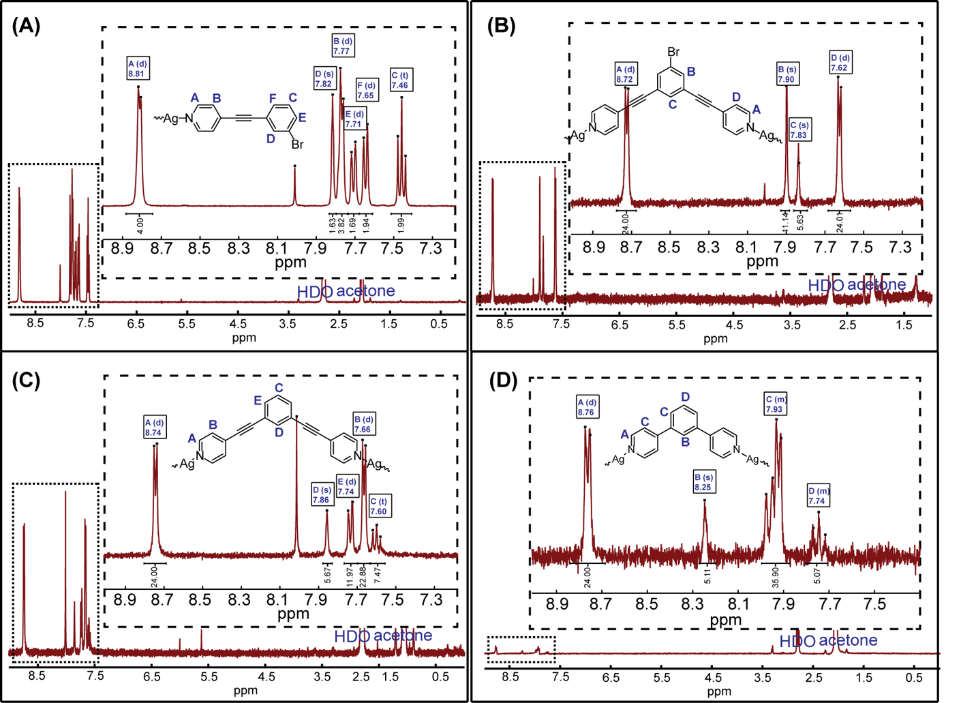


**Supplementary Figure 2**. ^1^H NMR spectra of **(A)** Ag**L1**_2_, **(B)** {Ag**L2**}_6_, **(C)** {Ag**L3**}_6_, and **(D)** {Ag**L4**}_6_ in acetone-d_6_


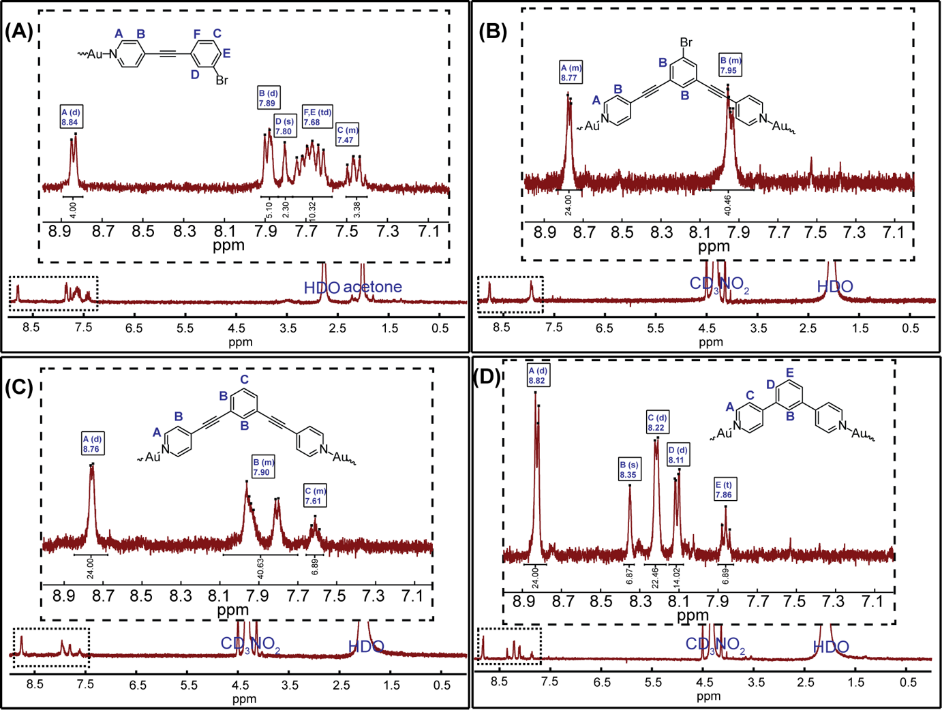


**Supplementary Figure 3**. ^1^H NMR spectra of **(A)** Au**L1**_2_, **(B)** {Au**L2**}_6_, **(C)** {Au**L3**}_6_, and **(D)** {Au**L4**}_6_ in acetone-d_6_

# K_f_ Determination using ^1^H NMR


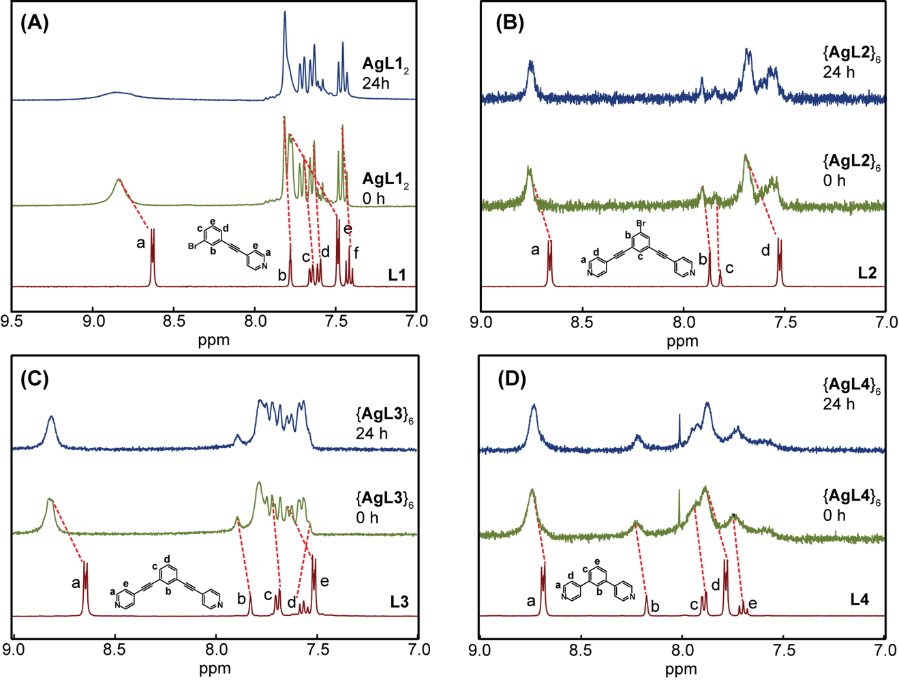


**Supplementary Figure 4**. ^1^H NMR spectra of **(A)** **L1** and Ag**L1**_2_, **(B)** **L2** and {Ag**L2**}_6_, **(C)** **L3** and {Ag**L3**}_6_, and **(D)** **L4** and {Ag**L4**}_6_ at 1:1 **L**:Ag in acetone-d_6_ from 0 to 24 h


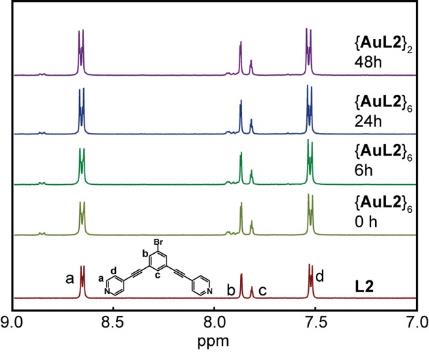


**Supplementary Figure 5**. ^1^H NMR spectra of **L2** and {Au**L2**}_6_ at 1:1 **L**:Au in acetone-d_6_ from 0 to 48 h


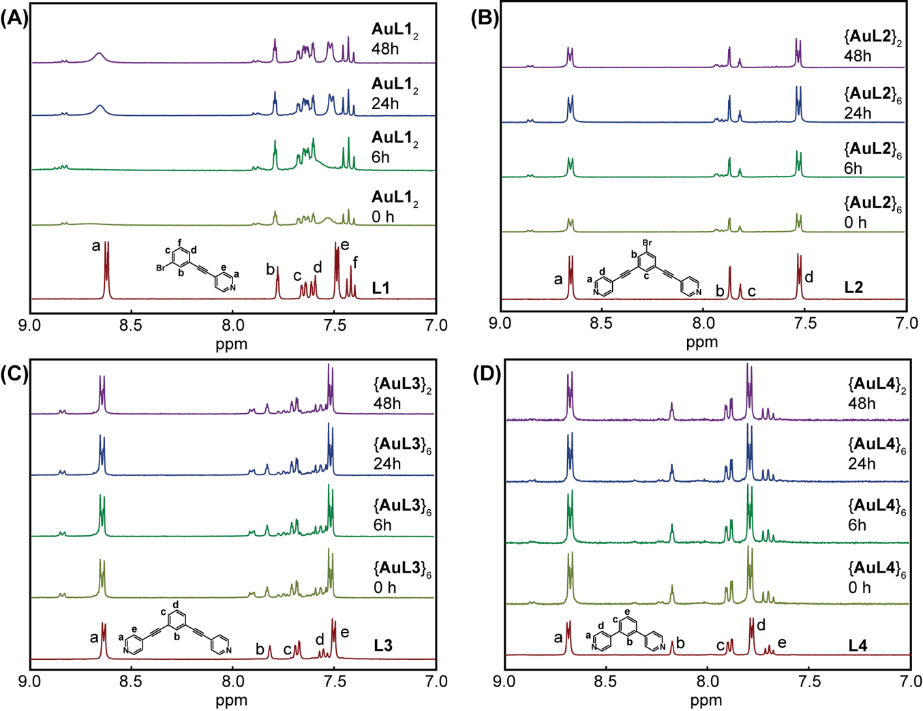


**Supplementary Figure 6**. ^1^H NMR spectra of **(A)** **L1** and Au**L1**_2_, **(B)** **L2** and {Au**L2**}_6_, **(C)** **L3** and {Au**L3**}_6_, and **(D)** **L4** and {Au**L4**}_6_ at 1:2 **L**:Au in acetone-d_6_ from 0 to 48 h


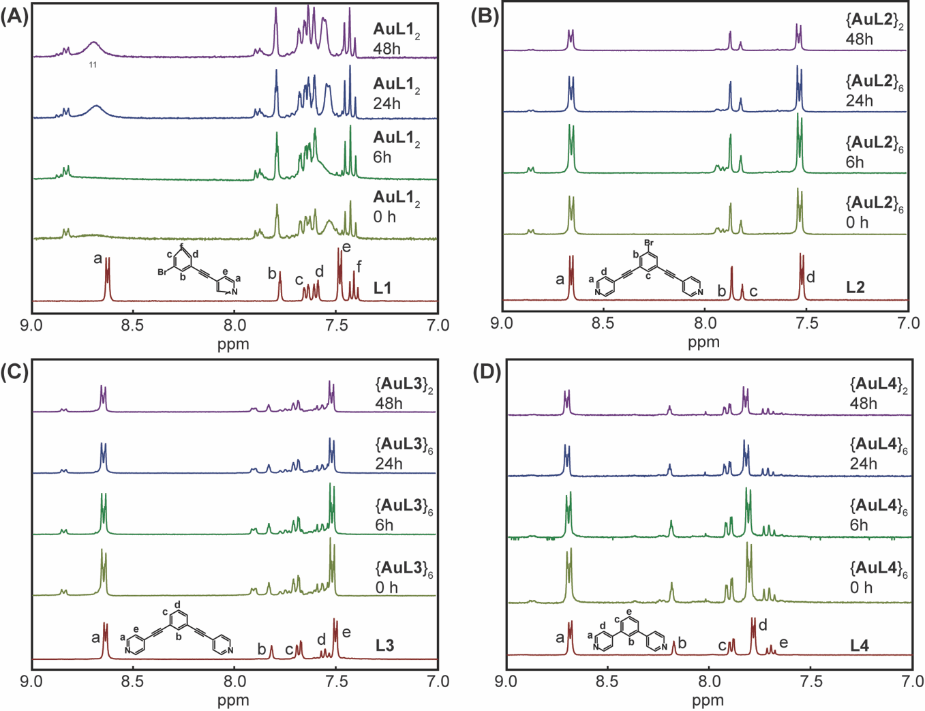


**Supplementary Figure 7**. ^1^H NMR spectra of **(A)** **L1** and Au**L1**_2_, **(B)** **L2** and {Au**L2**}_6_, **(C)** **L3** and {Au**L3**}_6_, and **(D)** **L4** and {Au**L4**}_6_ at 1:3 **L**:Au in acetone-d_6_ from 0 to 48 h

# FT-ICR MS Characterization


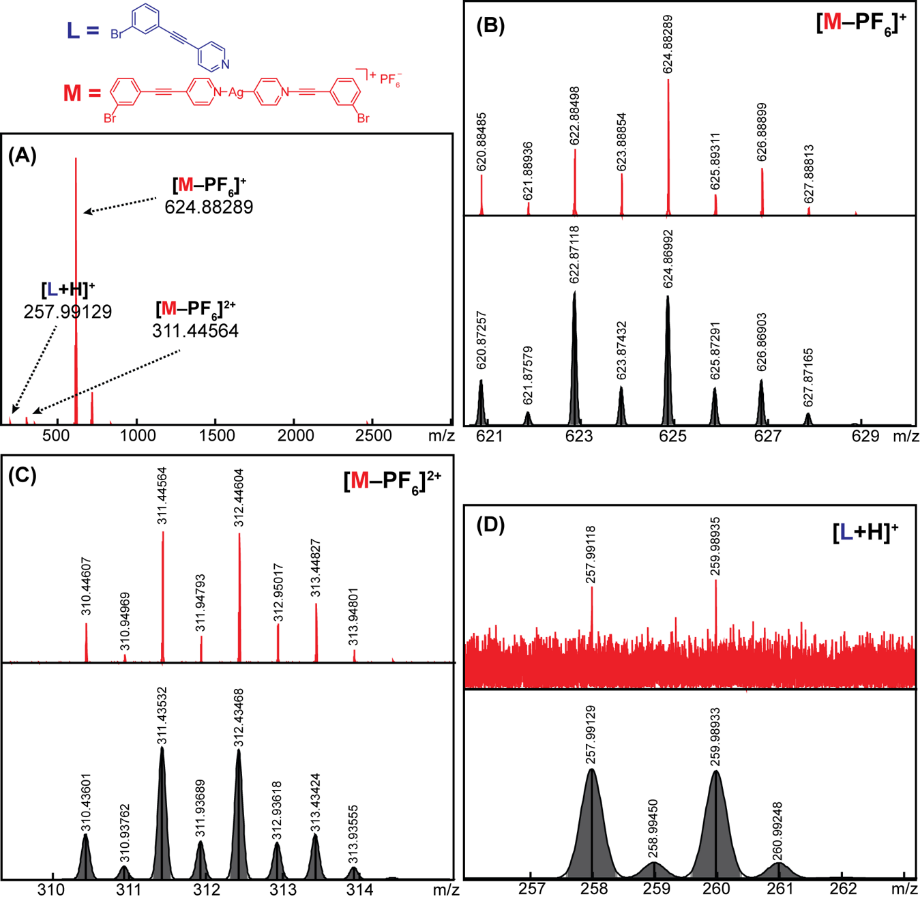


**Supplementary Figure 8**. ESI-FT-ICR MS **(A)** full spectrum, **(B** and **C)** spectra of intact Ag**L1**_2_ and **(D)** spectrum of **L1** (top = experimental; bottom = simulated)


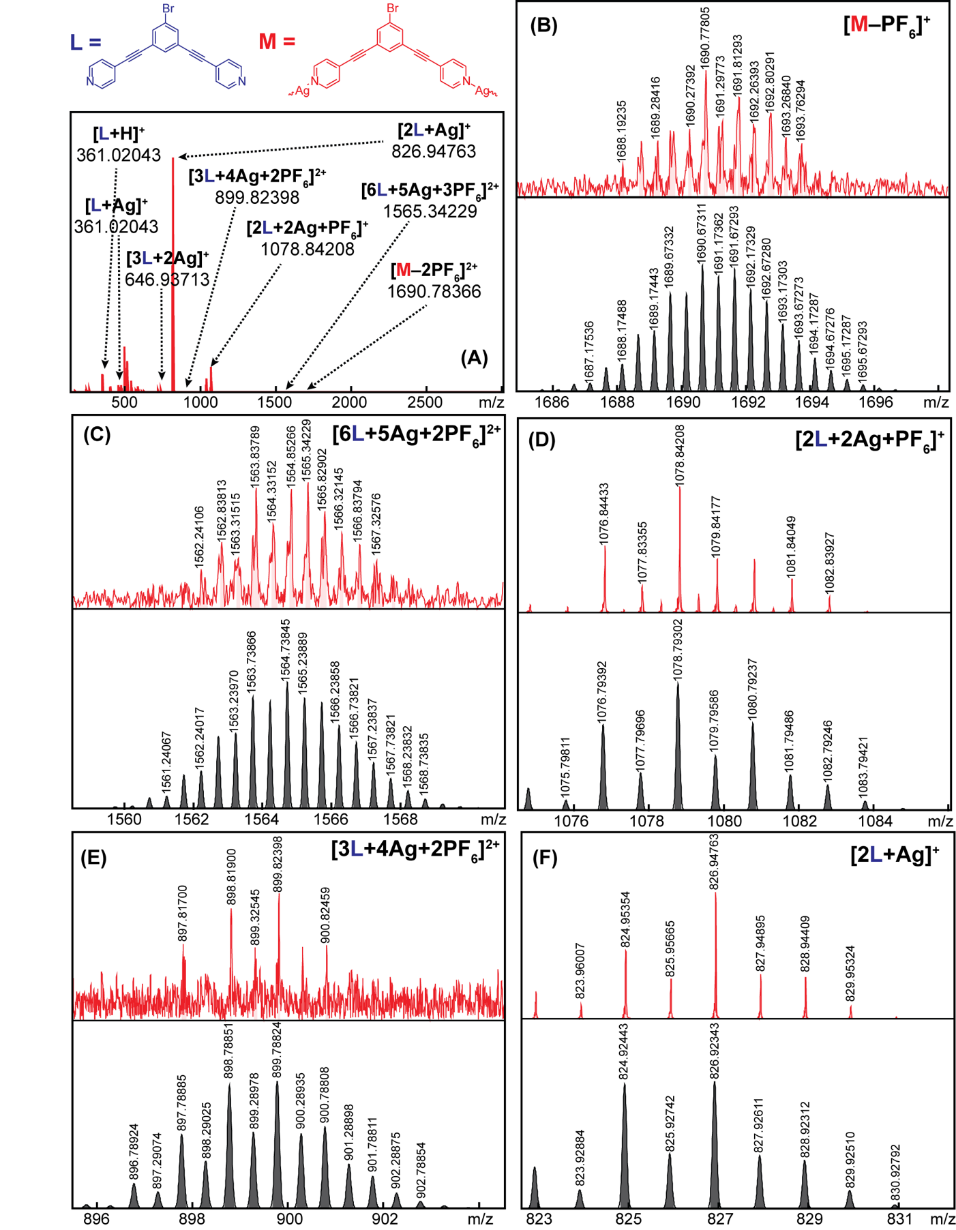


**Supplementary Figure 9**. ESI-FT-ICR MS **(A)** full spectrum, **(B)** spectra of intact, and **(C** to **F)** fragments of {Ag**L2**}_6_ (top = experimental; bottom = simulated)


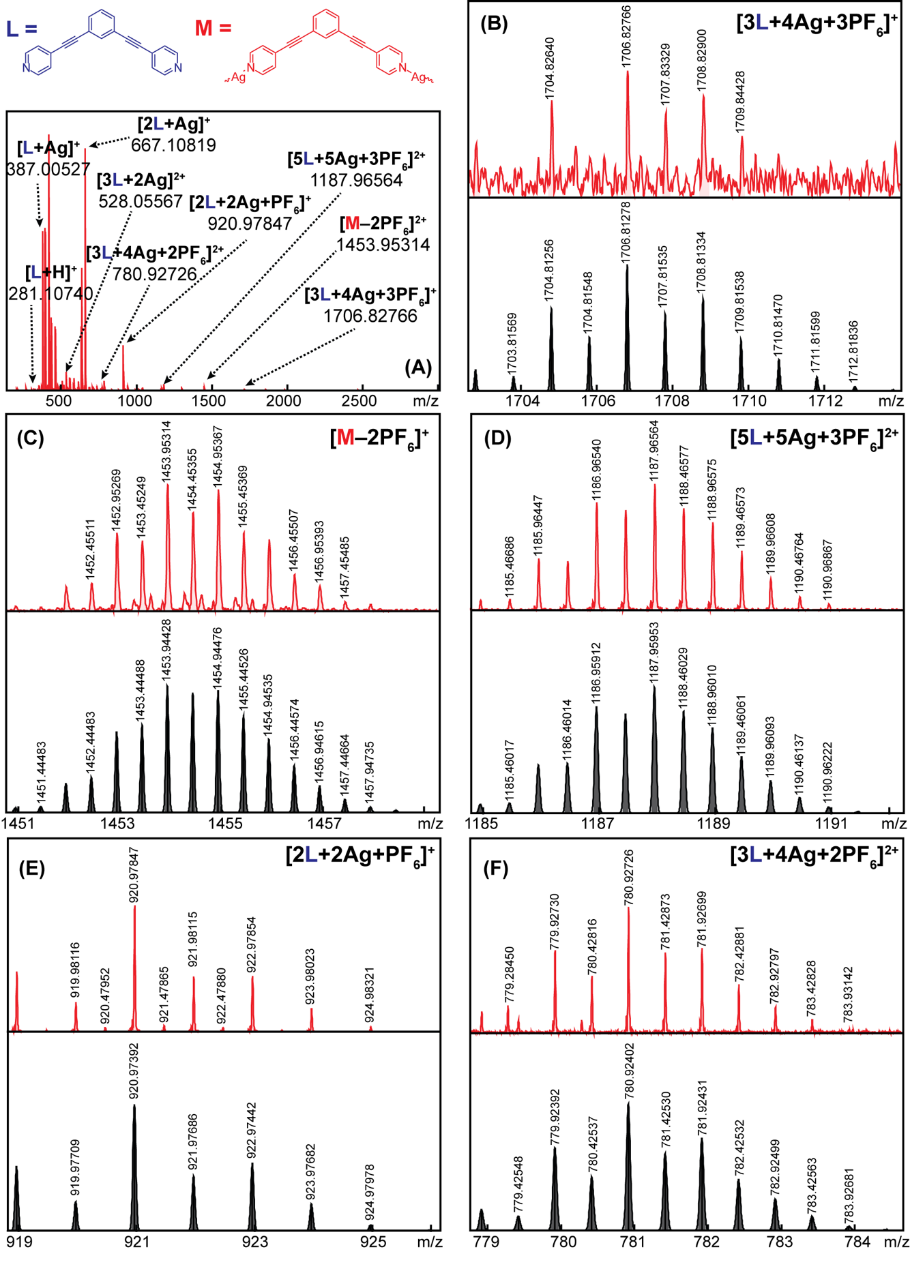


**Supplementary Figure 10**. ESI-FT-ICR MS **(A)** full spectrum, **(C)** spectra of intact, and **(B, D** to **F)** fragments of {Ag**L3**}_6_ (top = experimental; bottom = simulated)


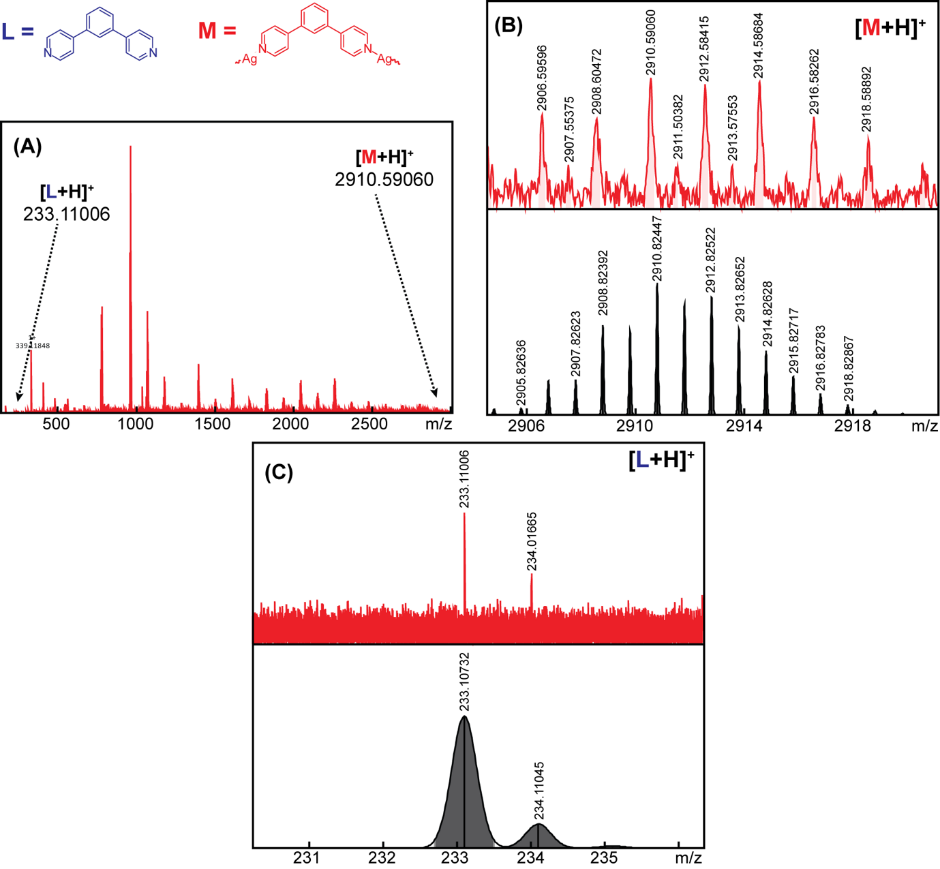


**Supplementary Figure 11**. LDI-FT-ICR MS **(A)** full spectrum, **(B** to **C)** spectra of {Ag**L4**}_6_ fragments (top = experimental; bottom = simulated)


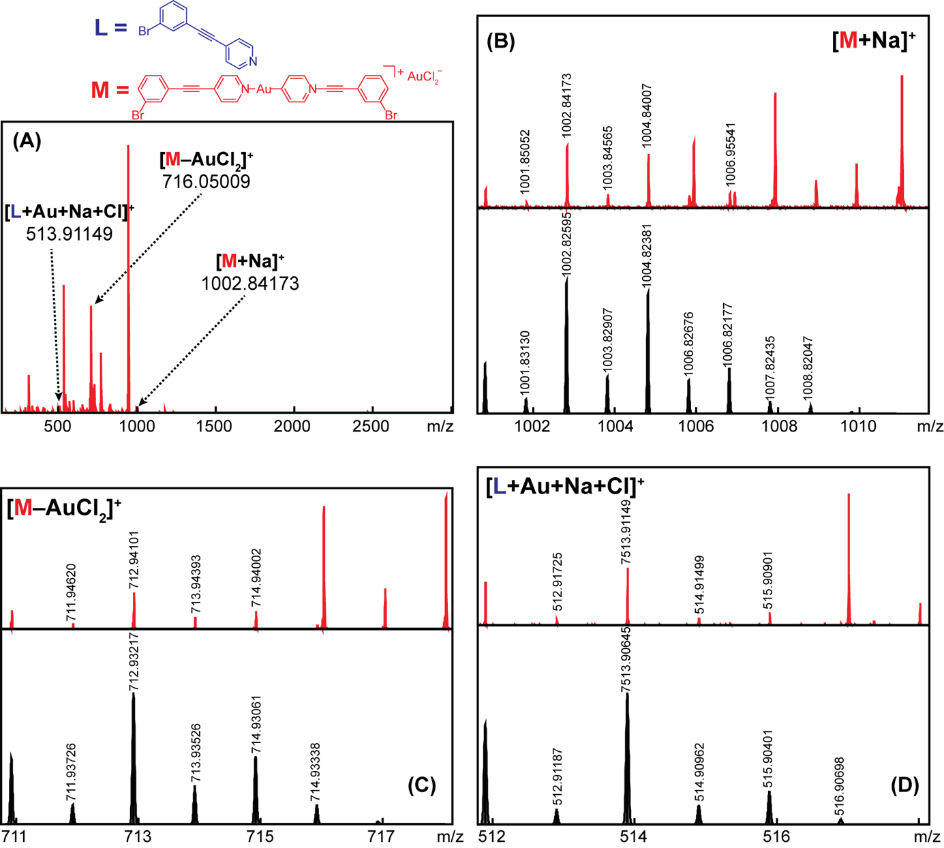


**Supplementary Figure 12**. ESI-FT-ICR MS **(A)** full spectrum, **(B** and **C)** spectra of intact and **(D)** spectrum of Au**L1**_2_ fragment (top = experimental; bottom = simulated)


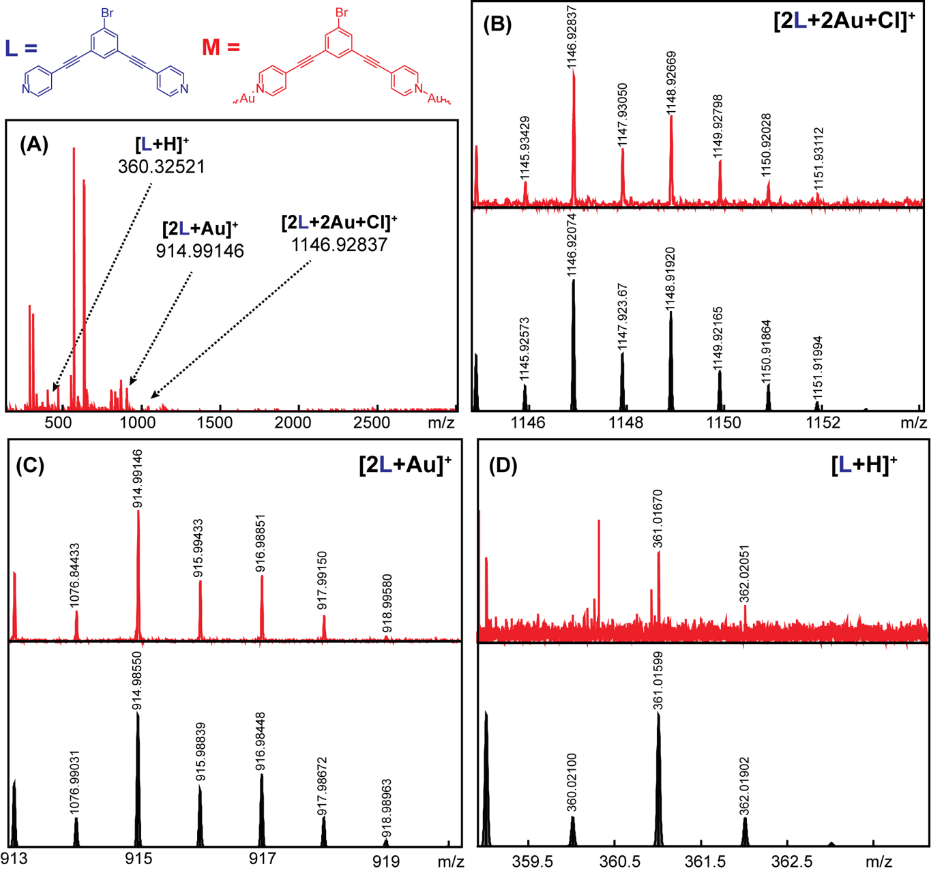


**Supplementary Figure 13**. ESI-FT-ICR MS **(A)** full spectrum, **(B** to **D)** spectra of {Au**L2**}_6_ fragments (top = experimental; bottom = simulated)


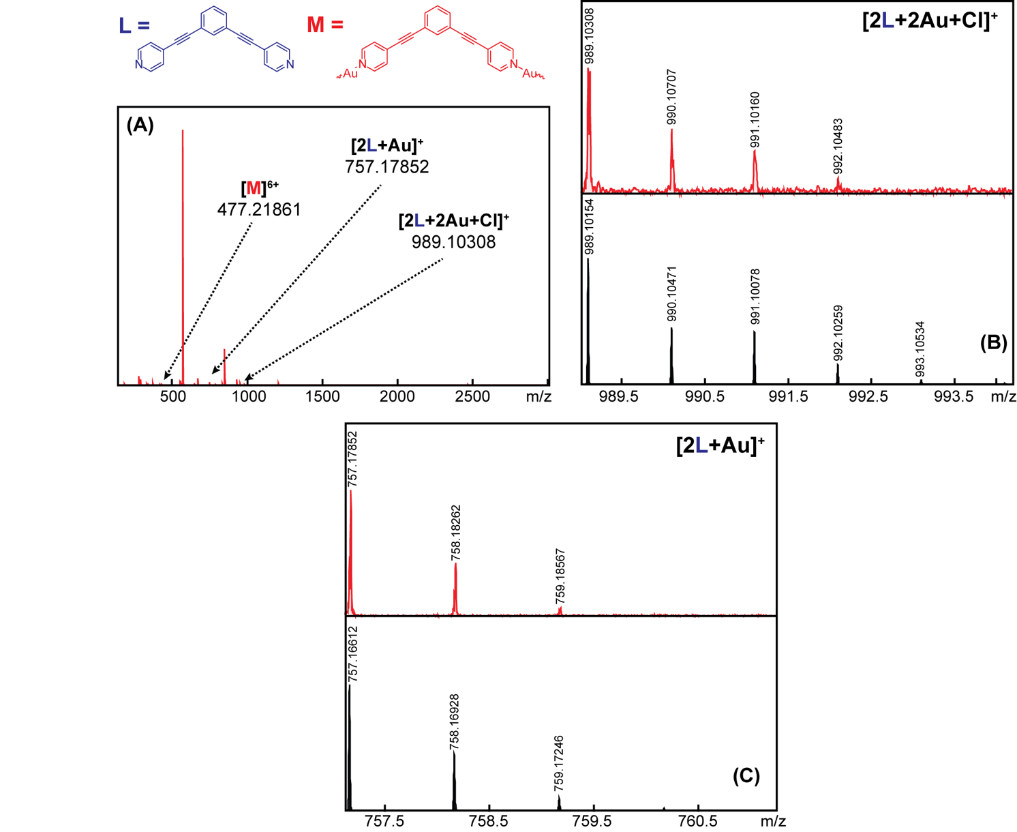


**Supplementary Figure 14**. ESI-FT-ICR MS **(A)** full spectrum and **(B** to **C)** spectra of {Au**L3**}_6_ fragments (top = experimental; bottom = simulated)


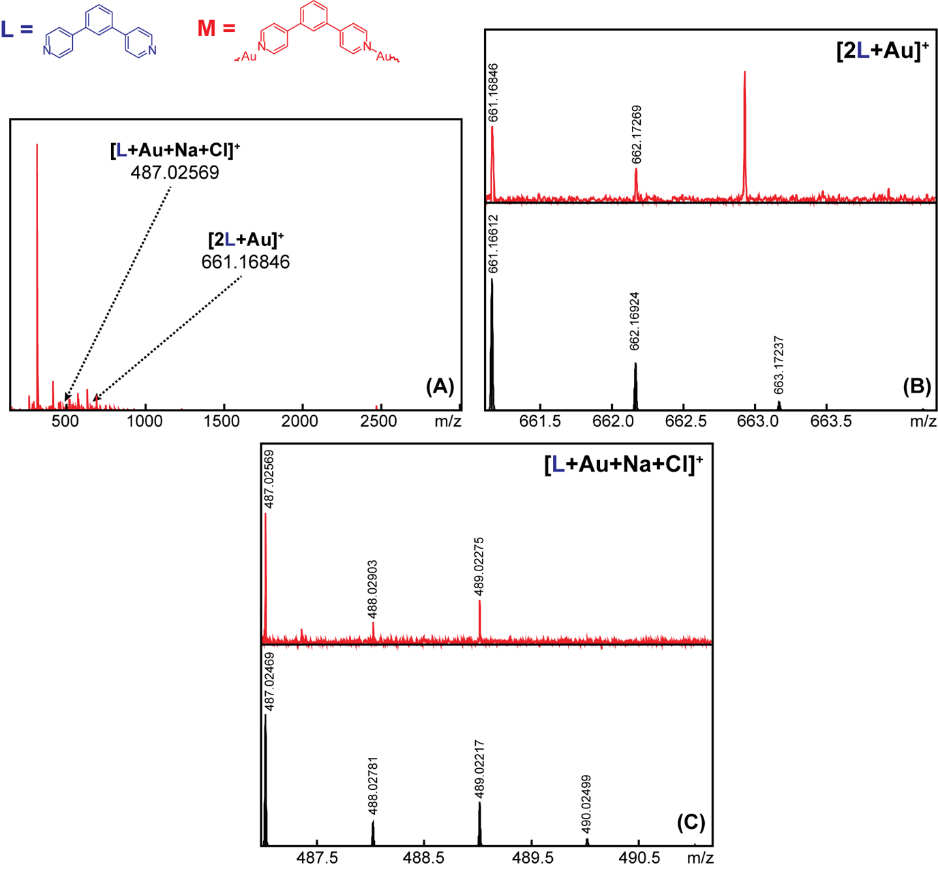


**Supplementary Figure 15**. ESI-FT-ICR MS **(A)** full spectrum, **(B** to **C)** spectra of {Au**L4**}_6_ fragments (top = experimental; bottom = simulated)

# Crystal Structure Details for AgL1_2_

Single crystals of Ag**L1**_2_ were grown from a CHCl_3_/MeOH solution at 4°C. The colorless crystals exhibited a long, needle-like, morphology. The datum crystal (0.04 x 0.06 x 0.30 mm^3^) was suspended in N-Paratone oil, mounted on a MiTeGen MicroLoop and cooled to 115 K. Data was collected on a Bruker D8 Venture diffractometer in a fixed-chi geometry, equipped with a Photon-100 CMOS area detector, Oxford Cryosystems cryostat, a molybdenum source (Mo, λ = 0.71073 Å), and a graphite monochromator.

Data collection proceeded routinely using a series of φ and ω scans to cover reciprocal space. Diffraction beyond 0.90 Å was not achieved, likely a result of the two short dimensions of the crystal; beyond this, all other metrics were reasonable. Data reduction was performed within APEX3,(Bruker, 2016) integration was performed using SAINT, semi-empirical multi-scan absorption correction was applied using SADABS, and the space group was determined using XPREP by examination of systematic absences, E2 statistics, and the successful refinement of the structure. Using Olex2 a structure solution was obtained using SHELXS via Direct Methods and least squares refined was carried out using SHELXL.(Sheldrick, 2008; Dolomanov et al., 2009; Sheldrick, 2015) Refinement was routine, rotational disorder was modeled for one the PF_6_^-^ counter ions. The chemically reasonable rigid bond restraints SIMU and DELU were applied to the entire structure given the rigid nature of the linear complex. ISOR restraints were used to isotropically refine anisotropic atoms in several cases. An EADP constraint was applied to two of the carbons atoms of the terminus phenylene ring to enforce similar atomic displacement parameters. This is chemically reasonable given the rigid nature of the phenylene ring. The resulting model refined well against the experimental data, and all refinement metrics fell within reasonable ranges.


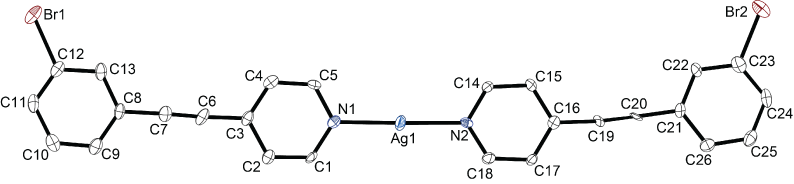


**Supplementary Figure 16**. Oakridge thermal ellipsoid plot (ORTEP) of Ag**L1**_2_ (CCDC No. 1879931). Probability level set at 50%, H-atoms, PF_6_^-^ counter ions, and CHCl_3_ solvents hidden for clarity.

**Table S1.** Crystallographic data for Ag**L1**_2_

| Empirical formula | C_26_H_16_AgBr_2_N_2_PF_6_·0.5CHCl_3_ |
| --- | --- |
| CCDC Number | 1879931 |
| Formula Weight (g/mol) | 828.75 |
| Temperature (K) | 115(2) |
| Crystal system | Monoclinic |
| Space group | P2_1_/*n* |
| *a* (Å) | 8.9335(5) |
| *b* (Å) | 41.270(2) |
| *c* (Å) | 15.7132(9) |
| α (°) | 90 |
| β (°) | 103.8680(17) |
| γ (°) | 90 |
| Volume (Å^3^) | 5624.4(6) |
| Z | 8 |
| ρ_calc_ (g cm^–3^) | 1.957 |
| μ (mm^–1^) | 3.819 |
| F (000) | 3208 |
| Crystal size (mm^3^) | 0.3 x 0.06 x 0.04 |
| Radiation | MoKα (λ = 0.71073) |
| 2Θ range for data collection (°) | 2.826 to 23.388 |
| Index ranges | –9 ≤ h ≤ 9, –46 ≤ k ≤ 46, –17 ≤ l ≤ 17 |
| Reflections collected | 8111 |
| Independent reflections | 6407 [R_int_ = 0.0724, R_sigma_ = 0.0608] |
| Data/restraints/parameters | 6407/923/753 |
| Goodness-of-fit on *F^2^* | 1.199 |
| Final R indexes [I>=2*σ*(I)] | R_1_ = 0.0697, wR_2_ = 0.1537 |
| Final R indexes [all data] | R_1_ = 0.0896, wR_2_ = 0. 1467 |
| Largest diff. peak/hole (e Å^–3^) | 1.67/–1.83 |

# Solid- and Solution-State Φ Determination


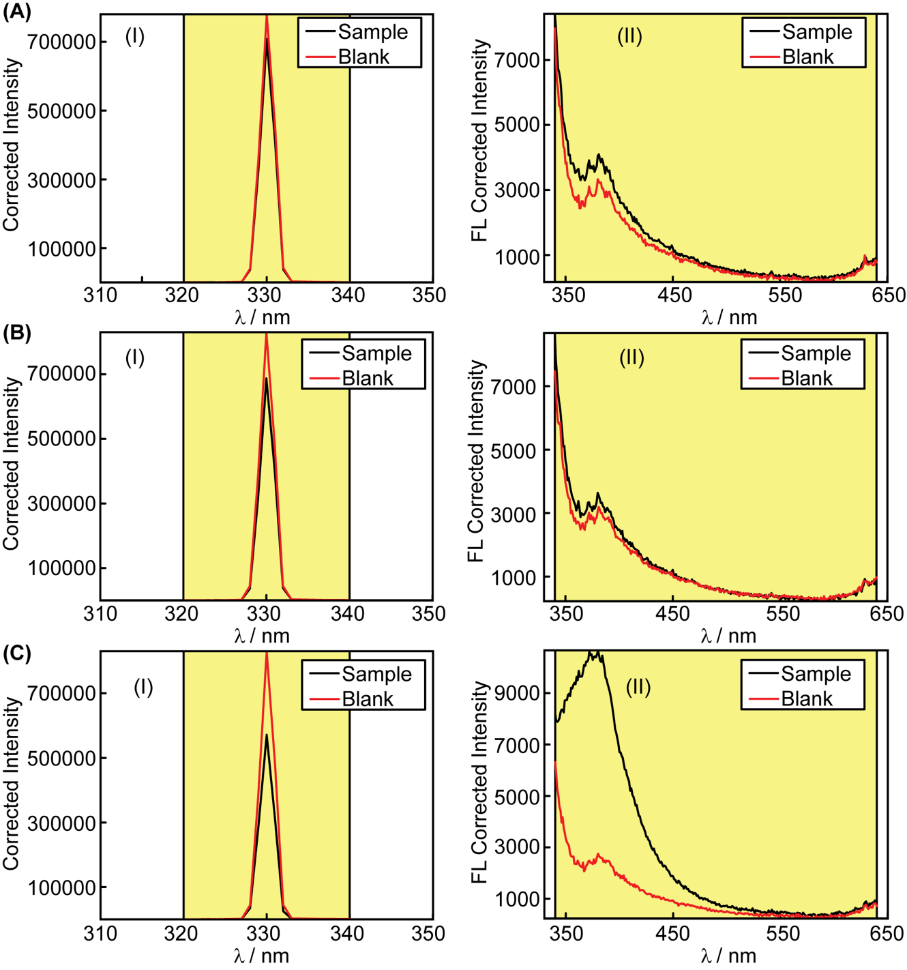


**Supplementary Figure 17**. Corrected light-sphere solution-state emission spectra in acetone of **(A)** 0.10 mM **L1**, **(B)** 0.10 mM **L2**, and **(C)** 0.10 mM **L3** at (I) 320–340 nm excitation and (II) 340–640 nm emission ranges


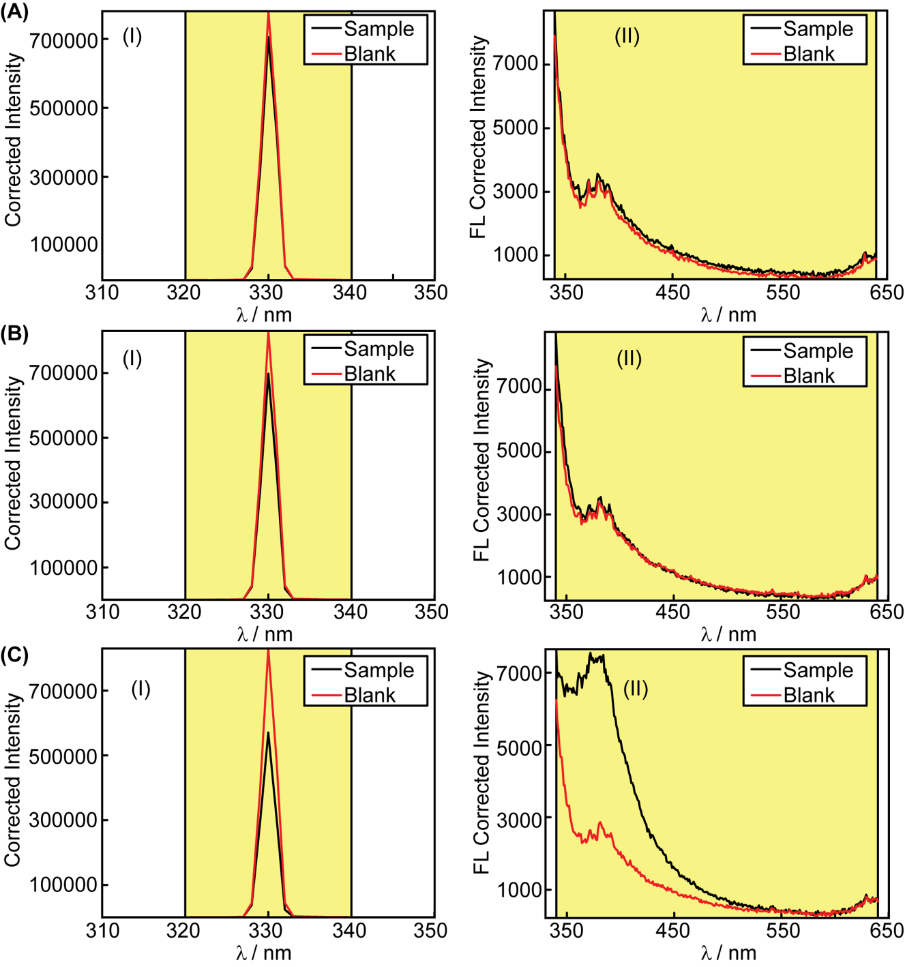


**Supplementary Figure 18**. Corrected light-sphere solution-state emission spectra in acetone of **(A)** 0.1 mM Ag**L1**_2_, **(B)** saturated {Ag**L2**}_6_, and **(C)** saturated {Ag**L3**}_6_ at (I) 320–340 nm excitation and (II) 340–640 nm emission ranges


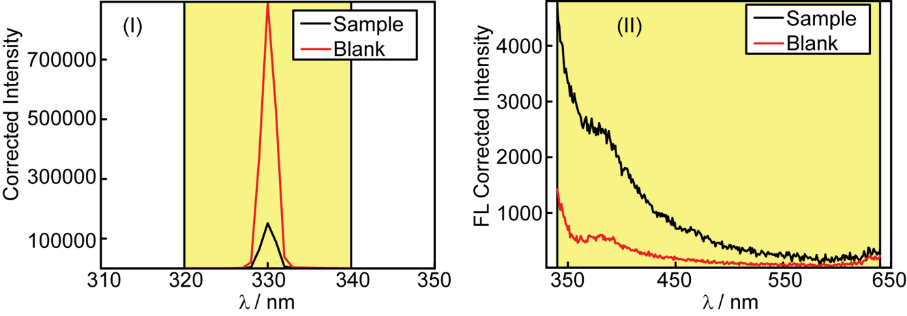


**Supplementary Figure 19**. Corrected light-sphere solution-state emission spectra in acetone of 0.10 mM Au**L1**_2_ (I) 320–340 nm excitation and (II) 340–640 nm emission ranges


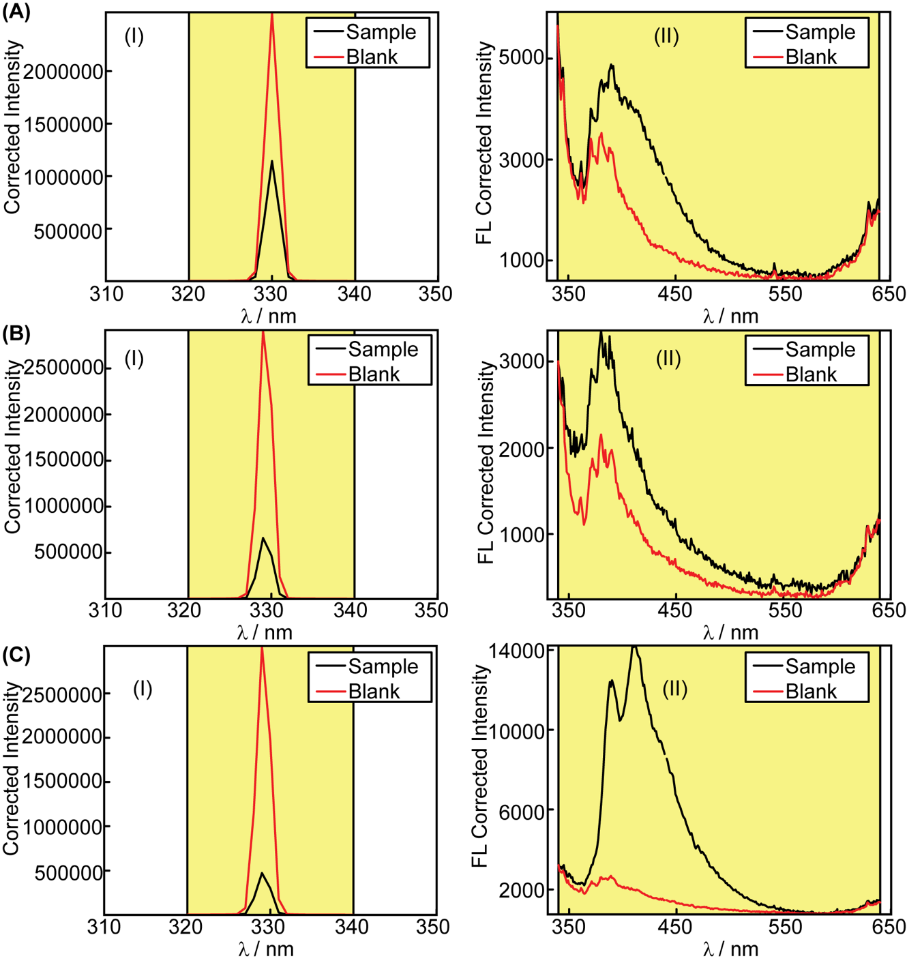


**Supplementary Figure 20**. Corrected light-sphere solid-state emission spectra of **(A)** **L1**, **(B)** **L2**, and **(C)** **L3** at (I) 320–340 nm excitation and (II) 340–640 nm emission ranges


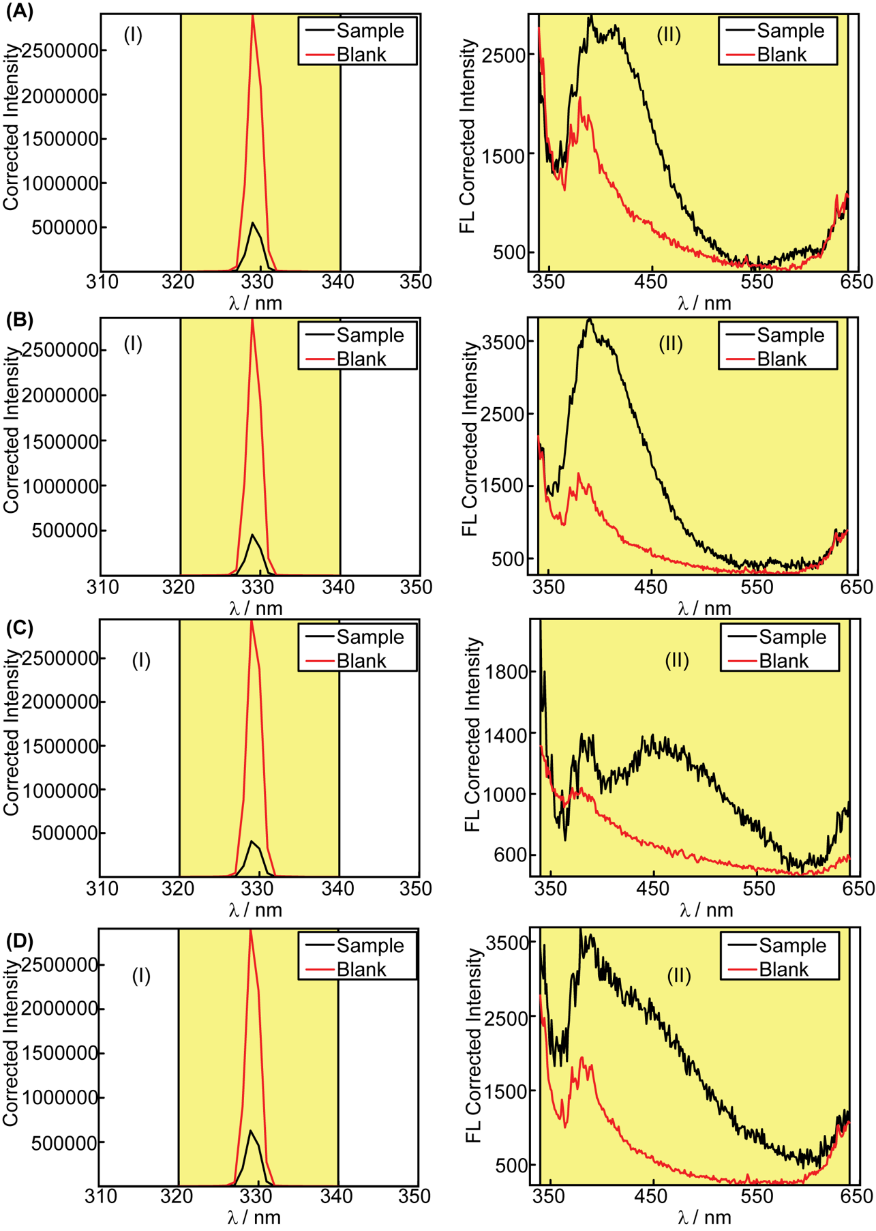


**Supplementary Figure S21**. Corrected light-sphere solid-state emission spectra **(A)** Ag**L1**_2_, **(B)** {Ag**L2**}_6_, **(C)** {Ag**L3**}_6_, and **(D)** {Ag**L4**}_6_ at (I) 320–340 nm excitation and (II) 340–640 nm emission ranges


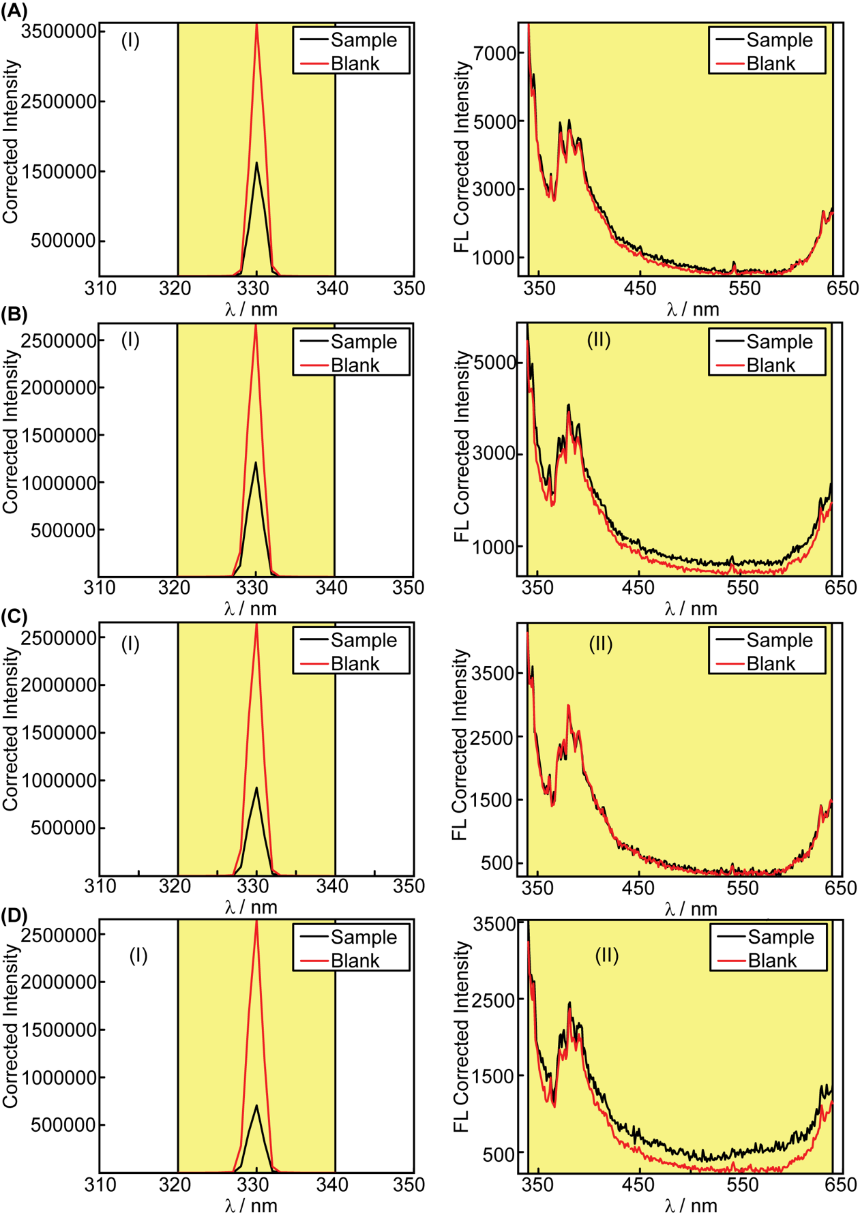


**Supplementary Figure 22**. Corrected light-sphere solid-state emission spectra **(A)** Au**L1**_2_, **(B)** {Au**L2**}_6_, **(C)** {Au**L3**}_6_, and **(D)** {Au**L4**}_6_ at (I) 320–340 nm excitation and (II) 340–640 nm emission ranges

# TCSPC Lifetime Determination


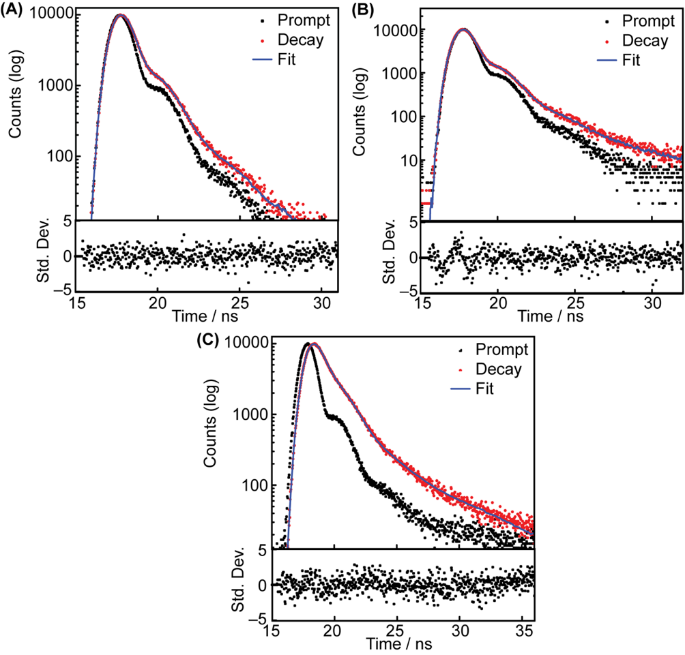


**Supplementary Figure 23**. TCSPC prompt traces, decay traces, best-fit models, and residuals of **(A)** 0.10 mM **L1**, **(B)** 0.10 mM **L2**, and **(C)** 0.10 mM **L3** in acetone


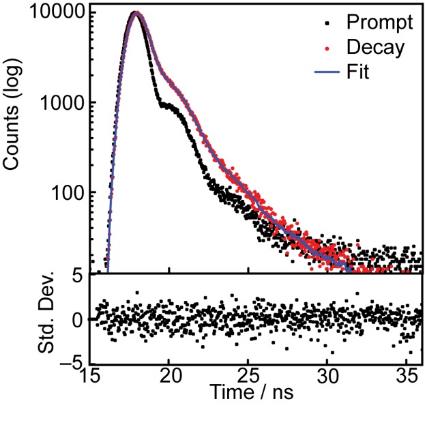


**Supplementary Figure 24**. TCSPC prompt trace, decay trace, best-fit model, and residual of saturated {Ag**L3**}_6_


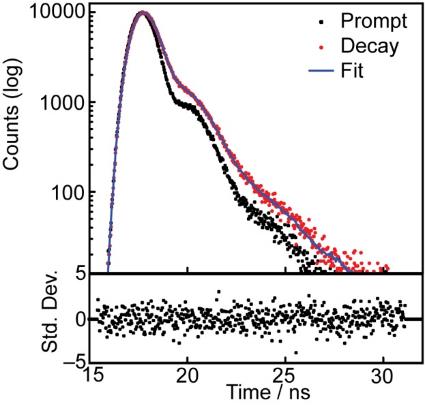


**Supplementary Figure 25**. TCSPC prompt trace, decay trace, best-fit model, and residual of 0.10 mM Au**L1**_2_

# References

Bruker (2016). "APEX3". (Madison, Wisconsin, USA.: Bruker–Nonius AXS Inc.).

Dolomanov, O.V., Bourhis, L.J., Gildea, R.J., Howard, J.A.K., and Puschmann, H. (2009). OLEX2: a complete structure solution, refinement and analysis program. *Journal of Applied Crystallography* 42(2)**,** 339-341. doi: doi:10.1107/S0021889808042726.

Lo, S.-C., Harding, R.E., Brightman, E., Burn, P.L., and Samuel, I.D.W. (2009). The development of phenylethylene dendrons for blue phosphorescent emitters. *Journal of Materials Chemistry* 19(20)**,** 3213-3227. doi: 10.1039/B820235D.

Sheldrick, G. (2008). A short history of SHELX. *Acta Crystallographica Section A* 64(1)**,** 112-122. doi: doi:10.1107/S0108767307043930.

Sheldrick, G. (2015). Crystal structure refinement with SHELXL. *Acta Crystallographica Section C* 71(1)**,** 3-8. doi: doi:10.1107/S2053229614024218.

Sun, B., Wang, M., Lou, Z., Huang, M., Xu, C., Li, X., et al. (2015). From Ring-in-Ring to Sphere-in-Sphere: Self-Assembly of Discrete 2D and 3D Architectures with Increasing Stability. *Journal of the American Chemical Society* 137(4)**,** 1556-1564. doi: 10.1021/ja511443p.

Uson, R., Laguna, A., Laguna, M., Briggs, D.A., Murray, H.H., and Fackler, J.P. (2007). "(Tetrahydrothiophene)Gold(I) or Gold(III) Complexes," in *Inorganic Syntheses*. John Wiley & Sons, Inc.), 85-91.

Wiesler, U.M., Berresheim, A.J., Morgenroth, F., Lieser, G., and Müllen, K. (2001). Divergent Synthesis of Polyphenylene Dendrimers:  The Role of Core and Branching Reagents upon Size and Shape. *Macromolecules* 34(2)**,** 187-199. doi: 10.1021/ma991519m.
